# Fossil climbing perch and associated plant megafossils indicate a warm and wet central Tibet during the late Oligocene

**DOI:** 10.1038/s41598-017-00928-9

**Published:** 2017-04-13

**Authors:** Feixiang Wu, Desui Miao, Mee-mann Chang, Gongle Shi, Ning Wang

**Affiliations:** 1 Key Laboratory of Vertebrate Evolution and Human Origins of Chinese Academy of Sciences, Institute of Vertebrate Paleontology and Paleoanthropology, Chinese Academy of Sciences, Beijing, 100044 China; 2 Biodiversity Institute, University of Kansas, Lawrence, KS 66045 USA; 3 Nanjing Institute of Geology and Palaeontology, Chinese Academy of Sciences, Nanjing, 210008 China; 4 College of Life Science, Beijing Normal University, Beijing, 100875 China

## Abstract

Understanding the Tibetan Plateau’s palaeogeography and palaeoenvironment is critical for reconstructing Asia’s climatic history; however, aspects of the plateau’s uplift history remain unclear. Here, we report a fossil biota that sheds new light on these issues. It comprises a fossil climbing perch (Anabantidae) and a diverse subtropical fossil flora from the Chattian (late Oligocene) of central Tibet. The fish, *Eoanabas thibetana* gen. et sp. nov., is inferred to be closely related to extant climbing perches from tropical lowlands in south Asia and sub-Saharan Africa. It has osteological correlates of a labyrinth organ, which in extant climbing perches gives them the ability to breathe air to survive warm, oxygen-poor stagnant waters or overland excursion under moist condition. This indicates that *Eoanabas* likewise lived in a warm and humid environment as suggested by the co-existing plant assemblage including palms and golden rain trees among others. As a palaeoaltimeter, this fossil biota suggests an elevation of ca. 1,000 m. These inferences conflict with conclusions of a high and dry Tibet claimed by some recent and influential palaeoaltimetry studies. Our discovery prompts critical re-evaluation of prevailing uplift models of the plateau and their temporal relationships with the Cenozoic climatic changes.

## Introduction

**Figure 1:**
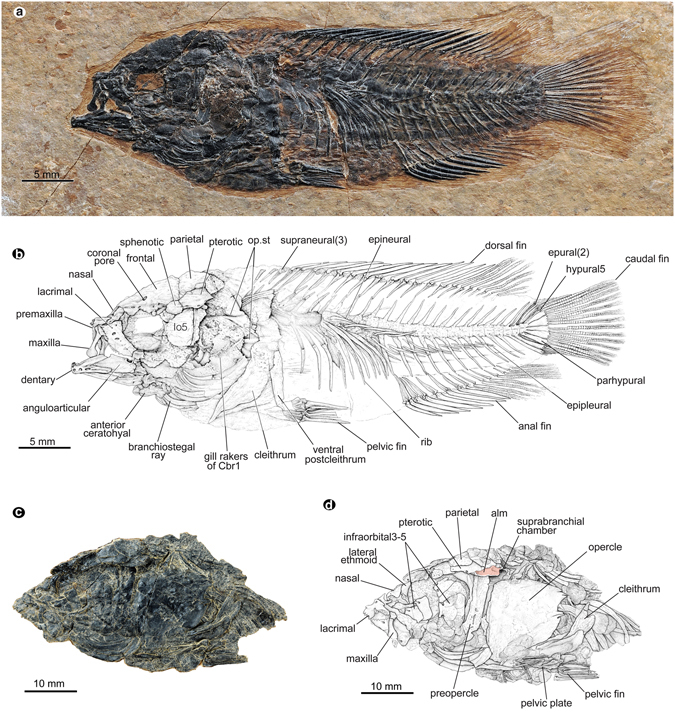
A new fossil climbing perch, *Eoanabas thibetana* gen. et sp. nov. from the upper Oligocene of central Tibet. It resembles its extant tropical relatives in having a labyrinth organ for air breathing and postocular contact organ in male fishes for stimulating the female during a mating clasp. (**a**) Photograph and (**b**) line drawing of holotype (IVPP V22782a), image horizontally rotated. (**c**) Photograph and (**d**) line drawing of the head of IVPP V18412a, red area in (**d**) representing muscular attachment facet. Abbreviations: alm, attachment facet of levator operculi muscle; Cbr1, ceratobranchial of first gill arch; op.st, V-shaped struts on inner side of opercles.

**Figure 2:**
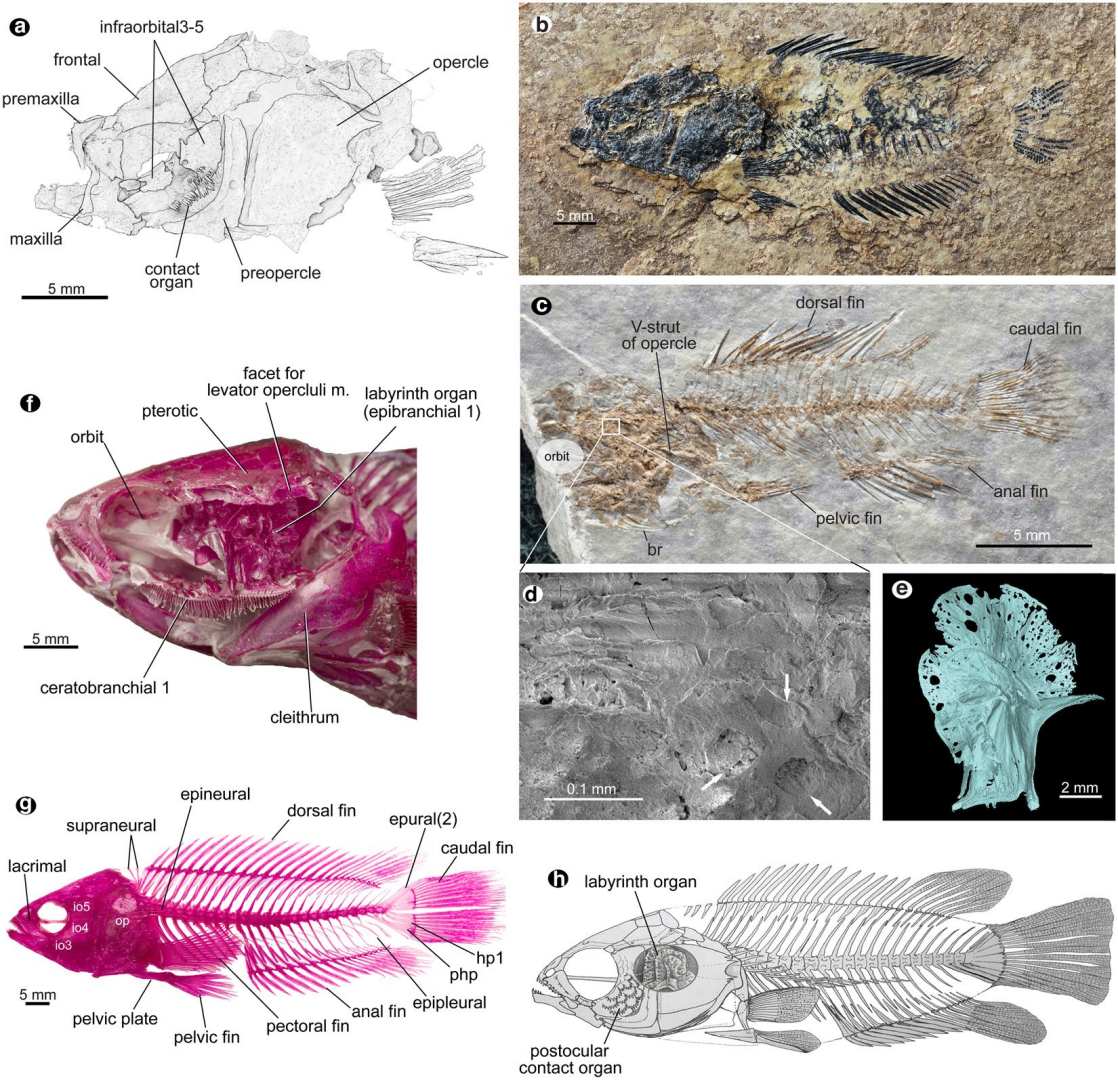
Fossil climbing perch, *Eoanabas thibetana* gen. et sp. nov. from the upper Oligocene of central Tibet. (**a**) Line drawing of the head of IVPP V18414a. (**b**) Photograph of IVPP V18414a. (**c**) Photograph of IVPP V18581a. (**d**) Scanning Electron Microscope (SEM) images of relics of labyrinth organ in (**c**), arrows pointing the pores on the lamellae. (**e**) Computerized tomography of labyrinth organ (lateral view) of *Anabas testudineus* (OP 435). (**f**) Cleared and stained head showing the labyrinth organ and associated structures of *Anabas testudineus* (collection no. OP 432). (**g**) Cleared and stained specimen of *Anabas testudineus* (collection no. OP 433). (**h**) Osteological restoration of *Eoanabas*, purported male; not to scale. Images in (**c**), (**d**) are horizontally rotated. Abbreviations: br, branchiostegal rays; hp1, hypural 1; m., muscle; php, parhypural.

**Figure 3:**
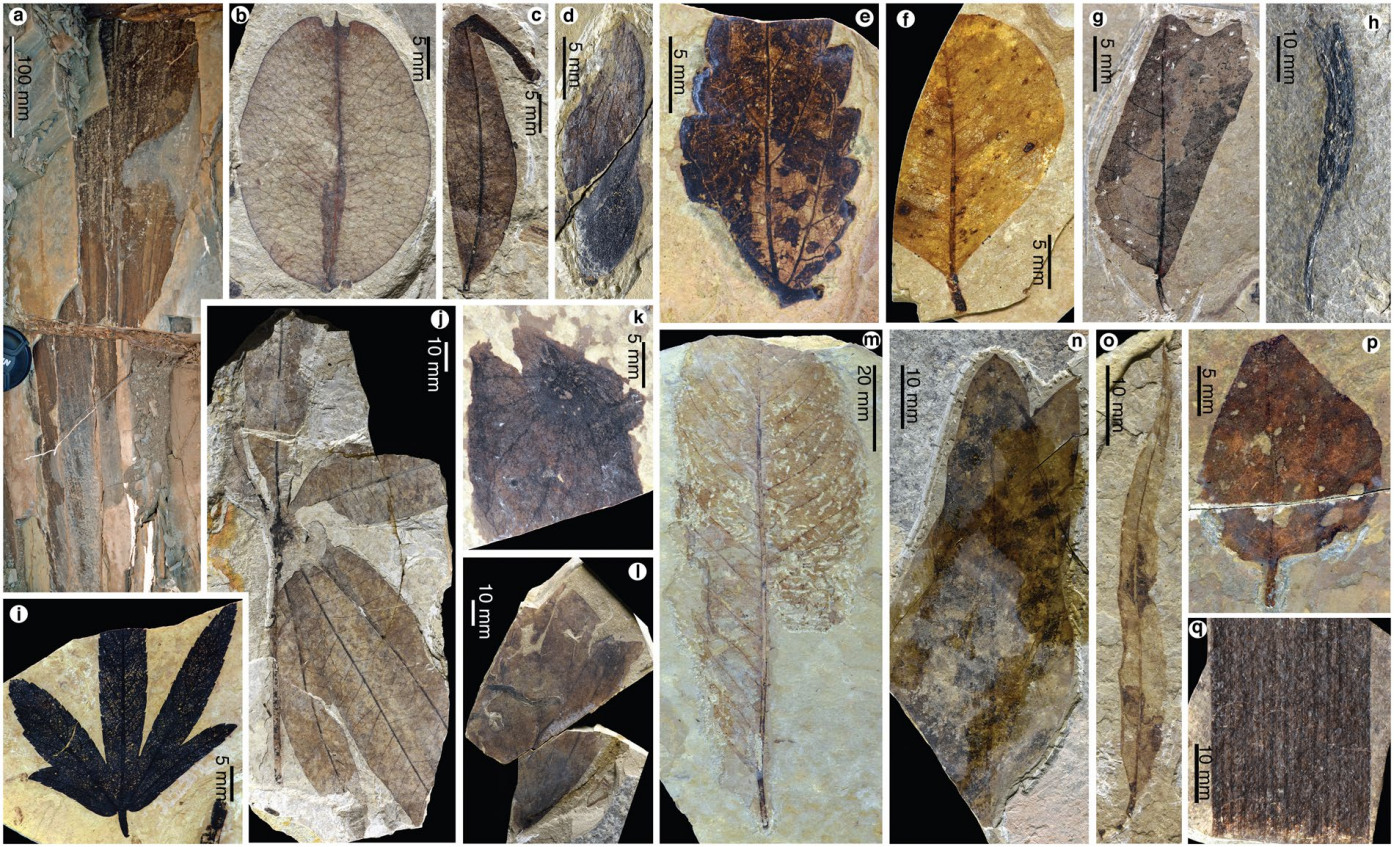
Plant megafossils associated with *Eoanabas*, showing the taxonomic and morphological diversity of the plant assemblage. (**a**) Palmate leaf of palm, IVPP B 2505. (**b**) Capsular valve of *Koelreuteria* sp., a golden rain tree, IVPP B 2506. (**c**) Leaflet of *Pistacia* sp., IVPP B 2508. (**d**) Winged fruit of *Cedrelospermum* sp., IVPP B 2510. (**e**) Undetermined toothed leaf, IVPP B 2519. (**f**) Undetermined entire-margined leaf, IVPP B 2518. (**g**) Undetermined toothed leaf, IVPP B 2527. (**h**) Infructescence of probable Araceae, IVPP B 2535. (**i**) Leaf of Araliaceae, IVPP B 2515. (**j**) Palmately compound leaf of *Handeliodendron* sp., with six leaflets, IVPP B 2513. (**k**) Leaf fragment of *Limnobiophyllum* sp., IVPP B 2514. (**l**) Leaf of *Exbucklandia* sp., IVPP B 2516. (**m**) Leaf of Magnoliales, IVPP B 2526. (**n**) Undetermined entire-margined leaf, IVPP B 2525. (**o**) *Populus* sp., IVPP B 2523. (**p**) Undetermined entire-margined leaf, IVPP B 2517. (**q**) Leaf fragment of *Typha* sp., IVPP B 2529.

The rise of the Tibetan Plateau is generally held responsible for the climatic cooling and monsoon intensification during the Cenozoic^1–3^. However, the history of the plateau has been fiercely debated, with conflicting uplift models proposed^4^. Some recent palaeoaltimetry studies, including those isotope-based ones^5–7^, suggest the establishment of near-present high elevations and harsh environment in the Tibetan interior since the Eocene or late Oligocene (~50–26 Ma)^5–9^ or some high and east-west trending mountain ranges in southern and northern plateau since Palaeocene^10^. Other tectonic models propose much younger uniform^11^ or northward stepwise uplift events since late Eocene^12^. Compared to the prevailing abiotic (tectonic, geophysical and geochemical, etc.) data, palaeobiological evidence bearing on timelines for uplift is scant, especially for the vast interior plateau^5–9^. Among the biotic proxies, plant megafossils, fishes and other vertebrates are environmentally sensitive and thus promising candidates for constraining the uplift of the plateau^13–22^. In addition to a fragmentary forelimb bone of an early Miocene fossil rhino^20^, only two late Oligocene to early Miocene fossil cyprinid (carps) species have been reported to date from the vast central area of the plateau. One is a primitive snow carp, *Plesioschizothorax macrocephalus* Wu et Chen^16, 19^, which has living relatives endemic to the plateau and its periphery^17, 18^. The other, *Tchunglinius tchangii* Wang et Wu, is a barbine carp, related to a lineage otherwise currently distributed in South Asia and Africa^21^. Both of these fishes were probably inhabitants of comparatively low altitudes^16–19, 21^. The plant megafossils from the interior of the plateau are even less common; there is but one barberry species formally reported from the Miocene Hox Xil basin in the northern plateau that possibly suggests an elevation ~2–3 km lower than modern-day levels^14^. Our recent discovery of a dozen well-preserved skeletons of a fossil climbing perch (Perciformes: Anabantidae) (Figs 1 and 2, Supplementary Figs 3–5) together with fifty exquisitely-preserved plant megafossils (Fig. 3) from the upper Oligocene (ca. 26~23.5 Ma) of the Nima and Lunpola basins in central Tibet (Supplementary Figs 1 and 2, and see Supplementary Information) is an important addition to the fossil record of the Tibetan Plateau. These fossils represent a biota that comprises ideal environmental indicators of warm and humid environment at low elevations.

The climbing perches (anabantid fishes) are closely related to kissing gouramies and fighting fishes^23–25^ in the perciform suborder Anabantoidei (labyrinth fishes)^25^. They are common in freshwaters in tropical Asia and sub-Saharan Africa^23, 24^. Their labyrinth organs are so large that they occupy a large portion of the gill chamber. In fact, their tissues are not sufficient to meet the respiratory needs, and they are therefore obligatory air-breathers^24^. The capability of accessary air breathing allows some of anabantids to move onto land under moist conditions, or even reputedly climb trees, thus earning the common name “climbing perch”^23^.

## Results

**Systematic Palaeontology of Fossil Climbing Perch**

Teleostei Müller, 1845

Anabantiformes *sensu* Wiley and Johnson, 2010

Anabantoidei *sensu* Lauder and Liem, 1983

Anabantidae Bonaparte, 1839

*Eoanabas thibetana* gen. et sp. nov.

**Etymology**. The generic name combines ‘*Eo-*’ (Greek, early/primeval) with ‘*Anabas*’, the type genus of Anabantidae from tropical Asia. The specific name refers to Tibet, China.

**Holotype**. IVPP V 22782, a complete skeleton, part and counterpart (Fig. 1a,b).

**Paratypes**. Sixteen specimens are designated as paratypes (Supplementary Information).

**Locality and Horizon**. Jiangnongtangga (type locality) and Songwori in south Nima Basin and Dayu in Lunpola Basin in central Tibet (Supplementary Figs 1 and 2). Middle-upper part of Dingqing Formation, late Oligocene (Chattian) (ca. 26~23.5 Ma)^6, 20, 26^.

**Diagnosis**. A labyrinth fish displaying anabantid characteristics including a posterior notch of the opercle bounded by spines, a V-shaped strut on inner side of opercle and six to nine anal-fin spines. It shares with Asian anabantids the following derived characters: broad infraorbitals 3–5 completely covering the cheek, a sensory canal pore just behind sphenotic/pterotic junction and pelvic plate lying flat; and it shares with African anabantids some derived characters, e.g., sensory canal opening in between the infraorbitals, supraorbital commissure of the sensory canal absent and male postocular contact organ present.

### Description

The body is oblong with long-based dorsal and anal fins comprising anterior spinous and posterior soft-ray sections, a thoracic pelvic fin anteceded by a spine, and a truncated caudal fin (Figs 1a,b and 2b,c,h, Supplementary Figs 3a,b,i,j, 4a–e, j–m and 5a,b,d–f).

The skull roof is smooth. The frontals carry paired coronal pores on the supraorbital sensory canals (Fig. 1a,b and Supplementary Fig. 3a,b) and, as in the species of African *Microctenopoma* ^24^, lack the supraorbital commissure. The circumorbital bones include a ventrally serrated lacrimal and broad infraorbitals 3–5 covering the cheek completely (Figs 1 and 2a, Supplementary Fig. 5a,b). Similarly large infraorbitals are only seen in *Anabas* (Fig. 2g); however, the sensory pores are open between these elements, a character known only in African anabantids^24^. Just posterior to the sphenotic/pterotic junction, the pterotic bears a sensory opening (Fig. 1 and Supplementary Fig. 5a,b), which is present in *Anabas* but not in African forms^24^. More posteriorly, it has a depressed attachment facet for the levator operculi muscle (Fig. 1c,d and Supplementary Fig. 5a,b) as in living anabantids^24^. Immediately ventral to this facet is the suprabranchial chamber (Fig. 1c,d) housing the labyrinth organ referred to below. The parasphenoid shaft is straight without oral or orbital processes (Supplementary Figs 3a,b,g and 4j–m), and the transverse process is likely present (Supplementary Fig. 3h).

The opercle has a round posterior notch bounded by two large spines or spine patches (Figs 1c,d and 2a,b, Supplementary Figs 3a,b and 5a,b) and a V-shaped strut on the inner side emerging from the articular socket with the hyomandibular (Figs 1a,b and 2c, Supplementary Figs 3a,b, 4k and 5a,b).

The maxilla is relatively short and stout with a clubbed posterior end (Figs 1 and 2a,b). Stout and recurved teeth occur on the anterior oral edges of the premaxilla and dentary (Figs 1a,b and 2a, Supplementary Figs 3f and 4k).

The ventral postcleithrum directly contacts the flat lying pelvic plate (Fig. 1a,b and Supplementary Fig. 3g), which resembles that of *Anabas*, but differs from the dorsally angled one in African forms^24^.

The long-based dorsal and anal fins show relatively low numbers of anal spines (7–9) and soft rays (7–9) in both fins (Figs 1a,b and 2b,c, Supplementary Figs 3a,b,i,j, 4a–e, j–m and 5a,b,d–f, and Supplementary Table 1). The truncated caudal fin has a principal fin-ray formula of I-7/7-I (Figs 1a,b and 2b,c, Supplementary Figs 3a,b,i,j, 4j–m and 5d,e and Supplementary Table 1). The parhypural firmly attaches to the compound vertebra (Fig. 1a,b and Supplementary Fig. 3a,b,i,j), whereas in living anabantids, it is detached and widely separated from the vertebra^24^ (Fig. 2g).

In one specimen of *Eoanabas*, layers of bony lamellae just above the branchial chamber (Fig. 2c and Supplementary Fig. 3g,h) show a cribrate structure (Fig. 2d) almost identical to that of the expanded plates of the labyrinth organ (modified first epibranchial)^24^ in living anabantids (Fig. 2e,f), suggesting the presence of a labyrinth organ also in the fossil fish (Fig. 2h).

Sexual dimorphism is exemplified by the postocular contact organ located immediately posteroventral to the orbit, a patch of scales with large ctenii (Fig. 2a,b and Supplementary Figs 3a,b,d and 5c) that are much larger than those of flank scales (Supplementary Fig. 3a,c,e). This organ is rare among perciform fishes^24^ and is seen in all African anabantids except for the species of *Microctenopoma* and *Sandelia capensis* ^24, 27^.

### The Plant Megafossils Assemblage Associated with *Eoanabas*

*Eoanabas* was found in the lacustrine depositional environments with several aquatic herbs (Fig. 3 and Supplementary Information) such as duckweed (*Limnobiophyllum*, Lemnoideae, Araceae) (Fig. 3k) and bulrush (*Typha*) (Fig. 3q), associated with a diverse lowland vegetation (Fig. 3 and Supplementary Information) including palms (Fig. 3a), golden rain trees (*Koelreuteria*) (Fig. 3b), *Pistacia* (Fig. 3c), Ulmaceae (Fig. 3d), Araliaceae (Fig. 3i), *Handeliodendron* (Fig. 3j), *Exbucklandia* (Fig. 3l) and aspens (*Populus*) (Fig. 3o). Among the 15 types of woody dicot leaves in this plant assemblage, only five types have a toothed leaf margin, whereas the remaining 10 types all have an entire margin (Supplementary Table 2). Regarding the leaf size^28^, microphyll leaves are dominant in the assemblage, with at least 10 morphotypes.

## Discussion

A phylogenetic analysis based on morphological data resolves *Eoanabas* as the earliest diverging lineage within the family Anabantidae, with its African relatives being monophyletic and sister to the Asian single genus *Anabas* (Supplementary Fig. 6). *Eoanabas* is also the oldest anabantid known so far, extending the geological record (previously represented only by three opercular bones from Pleistocene Java)^29, 30^ of this family for ca. 20 Ma and expanding the geographic distribution of this family to the middle Cenozoic central Tibet.

Living anabantids are confined mostly to the regions in Asia and Africa dominated by the tropical monsoon (Supplementary Fig. 7), with the greatest diversity in the rain forests of western and central Africa^24, 31^. They inhabit small bodies of water in lowlands in tropical Asia and sub-Saharan Africa^23, 24, 31^ (Supplementary Table 3), rarely higher than 1,000 m asl^31^. With few exceptions in the rivers of west Africa^24^, they prefer warm and periodically oxygen-poor stagnant waters, under the optimal temperature range of 18 to 30 °C (Supplementary Table 3). The key adaptations to such conditions are the labyrinth organ (Fig. 2f), a highly convoluted structure covered by richly vascularized epithelia^24^, and the resultant accessary air-breathing capability^24, 32^. Their air-breathing behaviour is facilitated by several muscles, including the levator operculi muscle^32^ that leaves a characteristic marked facet on the pterotics in living anabantids^24^. Interestingly, *Eoanabas* also has a labyrinth organ (Fig. 1d) and similar arrangement of the air-breathing muscle on the pterotics (Fig. 1c,d and Supplementray Fig. 5a,b). These strikingly similar characters appear physiologically specific and environmentally diagnostic^31^, and thus may indicate similar habitat conditions.

*Eoanabas* possesses a postocular contact organ in the males, which is unique to certain African anabantids^24, 27^. This structure indicates a typical anabantid spawning behavior. When the male embraces the female, it thrusts the spines of this organ into her abdomen to stimulate her to release eggs and ensure fertilization^27^. The similar reproductive behaviour strengthens the ecological similarities between *Eoanabas* and its living confamilial relatives.

The plant megafossil assemblage found with *Eoanabas* contains some taxa with living analogues (e.g., the palms and duckweed) commonly associated with the extant climbing perches (Supplementary Information), and hence reinforces the above palaeoenvironmental inference. Although the available material is insufficient to conduct a quantitative climatic reconstruction, the relatively high percentage (>60%) of the entire-margined types among the woody dicot leaves suggests a warm and humid climate (Supplementary Information). The dominance of microphyll leaves is consistent with this climatic inference, and appears to further suggest that the late Oligocene central Tibetan vegetation is more comparable to modern subtropical vegetation in Zhejiang, East China^33^. Based on the co-existing range of the living analogues of these fossil plants, this assemblage is assumed to grow most likely at an elevation of ca. 1,000 m asl (Supplementary Information).

Collectively, the ecological signal of *Eoanabas* and associated plant megafossils, as well as the co-existing fossil barbel fish *Tchunglinius* ^21^, suggests that warm and humid lowland habitats were present in the Tibetan interior ca. 26 Ma, probably at an elevation ~1,000 m asl, just like the environmental settings for modern climbing perches (Supplementary Fig. 8). Such a vibrant ecosystem represented by a spiny-rayed fish, barbel carp, and a diverse subtropical vegetation is in sharp contrast to that in the central Tibet today^15, 17, 18^. Yet the general lowland landscape might exhibit some topographic variation within the catchment area according to the contemporaneous pollen data of a mixed coniferous-broadleaved forest^26^.

Such palaeoenvironmental and palaeotopographic inferences will help us reevaluate our current understanding of the rise of the plateau and its environmental and climatic consequences. A warm and humid central Tibet requires a palaeogeographic setting allowing sufficient warm moisture to reach as far as the plateau interior at least up to the late Oligocene. As such, the current high-standing topographic features in southern Tibet might not exist then to block the southerly moisture from the ocean. This is in conflict with the notions that high Gangdese range has already been in place since the Palaeocene^10^ or the plateau had undergone northward stepwise uplift from late Eocene time onward^12^, but is compatible with the predictions of the rise of the Himalaya until the Neogene^34^ and newly proposed hypothesis for the oceanic Greater Indian Basin that would have maintained a deep tropical water mass between Asia and India throughout most of the Palaeogene^35^. Given the intrinsic environmental signals of the fossil climbing perch and the highly diversified sub-tropical plants, the establishment of a central Tibet with near-present elevation and climatic condition since early to late Eocene^5, 7–9^ or late Oligocene^6^ also seems highly problematic. Therefore, we caution against simple links between the rather early uplift of the plateau and the global climatic cooling during the early Cenozoic era^9^. The generally low elevation in plateau interior and probably more southern areas around the Palaeogene/Neogene transition is an important condition to test the existing tectonic models of plateau uplift. It is compatible with the inferences of either a period of un-roofing^2^ or the cease of the uplift process and a Tibet no higher than 1,000 m during ca. 30–20 Ma^11^, but contradicts the models for the constantly thickened crust in central Tibet since at least as early as the late Oligocene^6^ or even earlier^8, 9^. Our estimation of a fairly low, warm and wet central Tibet implies that this area has been elevated ~4,000 m to attain its current conditions since latest Palaeogene. In light of our fossil discovery, the geological history of the Tibetan Plateau is in need of critical re-evaluation.

## Methods

Fossils were prepared mechanically with sharp needles. The Recent material for comparison was cleared and stained according to the method of Bai^36^. Photographs of fossils were taken with Nikon except Fig. 2d and Supplementary Fig. 3f,h that were images produced using Hitachi S-3700N Scanning Electron Microscope (SEM) (working condition: Fig. 2d, 3 kV, 98 µA, Working Distance = 25.4 mm; Supplementary Fig. 3f, 5 kV, 99 µA, WD = 16.7 mm; Supplementary Fig. 3h, 3 kV, 101 µA, WD = 25.5 mm); and Fig. 2e that is an X-ray micro-computerized tomography. For the latter, the scanning was carried out at the Key Laboratory of Vertebrate Evolution and Human Origins, Chinese Academy of Sciences (CAS) using the 225 kV micro-computerized tomography (developed by the Institute of High Energy Physics, CAS. The specimen (partial labyrinth organ of *Anabas*) was scanned with beam energy of 90 kV and a flux of 100 μA at a detector resolution of 6.27 μm per pixel using a 360° rotation with a step size of 0.5° and an unfiltered aluminium reflection target. A total of 720 transmission images were reconstructed in a 2,048*2,048 matrix of 1,563 slices using a two-dimensional reconstruction software developed by the Institute of High Energy Physics, CAS. The three dimensional reconstructions were created in VG Studio Max (v2.0), and images of the reconstructions were exported from VG Studio and finalized in Adobe Photoshop and Adobe Illustrator. Line drawings were done under a Leica MZ8 microscope with a camera lucida attachment and further prepared using Adobe Photoshop CS4.

To conduct a phylogenetic analysis to assess the systematic position of *Eoanabas* within the family Anabantidae, we developed a data matrix including 165 soft tissue, behavioural, and skeletal morphological characters (Wu *et al*. 2016.nex, Wu *et al*. 2016.tnt). These morphological characters are updated from ref. 24 and include 33 newly defined and other characters adopted from references elsewhere (for details see Appendix section below). Of the total 21 taxa, *Badis* and kissing gouramy *Helostoma* were designated as outgroups. The ingroup comprises fossil *Eoanabas thibetana* and 18 living anabantid species and species assemblages according to ref. 24. The data matrix was edited in WinClada 1.00.08^37^ and saved in the NEXUS format (Wu *et al*. 2016.nex) and TNT format (Wu *et al*. 2016.tnt). Additionally, to further test the phylogenetic position of the fossil taxon, we also tried the backbone-constrained search with the relationships based on Rüber *et al*.’s gene matrix^25^ of the extant taxa involved in this study used as backbone (datamatix: Wu *et al*. 2016 backboned.tnt). Parsimony analysis was undertaken using TNT, Tree analysis using New Technology, a parsimony analysis program supported by the Willi Hennig Society^38^. The analyses were conducted using a traditional search strategy, with default settings except for the following: 10,000 maximum trees in memory and 100 replications. Bremer support values were generated in TNT by applying the ‘New Traditional Search’ using TBR and collecting suboptimal topologies with 100 replicates. All characters were treated as unordered, and weighted equally.

## Electronic supplementary material

Supplementary Information

Dataset 1

Dataset 2

Dataset 3
