# Supplementary material for: Fossil climbing perch and associated plant megafossils indicate a warm and wet central Tibet during the late Oligocene
Source: Sci Rep. 2017 Apr 13;7:878. doi: 10.1038/s41598-017-00928-9 (PMC5429824; doi:10.1038/s41598-017-00928-9)
Supplement: Supplementary file 1 — Supplementary Information [file 41598_2017_928_MOESM1_ESM.doc]

**Supplementary Information for “Fossil climbing perch and associated plant megafossils indicate a warm and wet central Tibet during the late Oligocene”**

Feixiang Wu^1^, Desui Miao^2^, Mee-mann Chang^1^, Gongle Shi^3^, Ning Wang^4^

*^1^ Key Laboratory of Vertebrate Evolution and Human Origins of Chinese Academy of Sciences, Institute of Vertebrate Paleontology and Paleoanthropology, Chinese Academy of Sciences, Beijing 100044, China; ^2^ Biodiversity Institute, University of Kansas, Lawrence, KS 66045, U.S.A.; ^3^ Nanjing Institute of Geology and Palaeontology, Chinese Academy of Sciences, Nanjing 210008, China; ^4^ College of Life Science, Beijing Normal University, Beijing 100875, China*

1. **Geographical and Geological Background**

The Tertiary Nima Basin in central Tibet (Supplementary Fig. 1a) covers an area of about 3,000 km^2^ on the current average elevation of ~5,000 m asl with the annual mean temperature of ~ -1.0 ℃ and the annual mean precipitation of 150~210 mm^39^. It is an east-west trending rift basin located approximately on the surface trace of the Bangong-Nujiang suture between the Qiangtang and Lhasa terranes, with its fill resting on a Jurassic-Cretaceous marine succession containing deformed flysch and mélange^40−42^ (Supplementary Fig. 1b). About 250 km to the east of the Nima Basin is the Tertiary Lunpola Basin, which is closely similar to the Nima Basin in terms of age, sedimentary fill, and developmental history^42^. The Lunpola Basin is also a rift basin and located in the Bangong-Nujiang suture, which extends east-west for a distance of ca. 200 km and a width of 15-20 km with present elevations of ca. 4,600-5,040 m asl^26^. The Cenozoic deposits in this basin, up to 4,000 m thick, comprise mainly the Niubao Formation in the lower part and the Dingqing (Dingqinghu) Formation in the upper part. The Niubao Formation is characterized by reddish clastic deposits, dominated by mudstone, sandstone, and gravel and was assigned an age of Palaeocene to Eocene^26^. The Dingqing Formation is about 1,000 m thick and is characterized by lacustrine fine-grained greyish mudstone, fine siltstone, and limestone. The age of Dingqing Formation has been controversial^26^ and according to a recent geochronological study^26^ and mammal fossil evidence^20^ it can be assigned as late Oligocene to early Miocene. The fossil sites in the Nima Basin are located in its south margin exposed in the frontal ranges south to the Dagze Lake, Cuozheluoma Town and Saibu Lake (Supplementary Fig. 1b). The southern margin of this basin is marked by the regionally extensive, southward-dipping Gaize-Seling Co and Nima thrusts, with the northernmost exposures of the marine Aptian-Albian Langshan Formation in the hanging wall^40−42^ in the western part (Supplementary Fig. 1b). The Tertiary lithological unit including the fossiliferous strata was informally designated as Nima Redbed unit^40^ and defined as the Upper Cretaceous Jingzhushan Formation^39^; however, some recent studies based on biotic evidence and geochronological data confirmed that it is mid-Cenozoic in age^6,40−42^. The fossiliferous layers lie within the strata that yielded biotite Ar^40^/Ar^39^ age of ca. 26-23.5 Ma^40,41^, indicating a late Oligocene age and thus comparable to the middle to upper Dingqing Formation in the nearby Lunpola Basin.

The fossil material of the Nima Basin came from two sections of the same stratigraphical unit corresponding to the middle part of the Dingqing Formation^42,43^: one is named Jiangnongtangga section (31°47’ 43’’ N, 87°45’ 39’’ E) (Elevation 4,810 m asl) and the other is Songwori section (31°54’ 47’’ N, 88°06’46’’ E) (Elevation 4,794 m asl) (Supplementary Fig. 1b). The Jiangnongtangga section is adjacent to the sampled section 1DC in refs. 6, 40, 41 and in the south flank of the E-W-trending Nima syncline designated in references above along with sections 2DC, 4DC therein (Supplementary Fig. 1b). The Songwori section is the sampled section investigated in ref. 39. The sediments of the strata of these two sections comprise thinly-bedded greyish green mudstones and sheet-like calcareous shales inter-bedded with mudstones, sandstones, and limestones^39,42^ (Supplementary Fig. 2).

The fossil site (32°01’29’’ N, 89°46’19’’ E) (Elevation 4,683 m asl) from the Lunpola Basin is the Dayu section, which is situated near the village Tangnu in the local geological map^43,44^, several kilometers northwest to the sampled section (middle to upper Dingqing Formation, Upper Oligocene-Lower Miocene) in ref. 20 and a dozen kilometers east to the sampled section in ref. 26, respectively (Supplementary Fig. 1c). The strata yielding the fossil material consist mainly of greyish green and red mudstones interbedded with limestones and shales, and correspond to the sampled section in ref. 26, which is late Oligocene to earliest Miocene in age.

1. **Palaeontological remarks**

The Tertiary fossil fishes from the Jiangnongtangga section in the Nima Basin were first reported by Wang et al.^45^. Although there are photos of two fishes printed in that article, they were mistakenly identified under the insect family Hygrobiidae^45^. On the basis of those pictures we are certain that the fishes are not perciforms, but most probably are cyprinids (carps). Mainly on the basis of palynological analysis, the authors suggested the age of the fish-bearing deposits as the Oligocene to Miocene^45^. The investigations in the Nima Basin led by Institute of Vertebrate Paleontology and Paleoanthropology, CAS since 2010 have collected numerous fossils from the above-mentioned fossil sites, including fishes, insects, and plants, etc. Of the fishes, a cyprinid taxon, *Tchunglinius tchangii* that supposedly has affinity with the South Asian and African cyprinid *Puntius* was recently published^21^. And in the Lunpola Basin, the vertebrate fossils so far reported include only a primitive snow carp *Plesioschizothorax macrocephalus*^16,19^ and a rhinocerotid mammal (*Plesiaceratherium* sp.) represented by a fragmentary humerus bone^20^. Since 2009, we have conducted many explorations in the Lunpola and Nima Basins and collected some mammalian bones, hundreds of fossil fishes, plants and insects from the studied locality here.

**Coexisting fossil plant assemblage and its palaeoenvrionmental implications**

Totally 50 plant megafossil specimens were collected from the Dingqing Formation of the Dayu (Lunpola Basin) and Jiangnongtangga (Nima Basin) sections along with *Eoanabas*, representing one of the best-preserved plant fossil assemblages known in Tibet*.* Botanical affinities of the fossils were determined based on extensive comparison with extant plants. The fossil assemblage consists of leaves of 16 nonmonocot angiosperms and 7 monocots, and 5 types of fruits; some of them are quite exquisitely preserved (Fig. 3). The recognizable fruits include capsular valve of the golden rain trees (*Koelreuteria* Laxm., Sapindaceae) and winged fruits of *Cedrelospermum* Saporta, an extinct genus of the family Ulmaceae. Leaves hitherto identified to extant taxa below or at the family level include the palm, *Handeliodendron* Rehder (Hippocastanaceae), *Pistacia* L. (Anacardiaceae), *Exbucklandia* R. W. Brown (Hamamelidaceae), *Limnobiophyllum* (Lemnoideae, Araceae), *Typha* L. (Typhaceae), Araliaceae, Bignoniaceae and Lauraceae. All these genera except *Cedrelospermum* still exist in South China^46−53^. Fossils of palms^52^, *Koelreuteria*^53^, *Cedrelospermum*^54^, *Typha*, and *Pistacia*^55^ are known from the Palaeogene and Neogene of Europe, East Asia, and North America; fossils of *Exbucklandia* are only known from the Oligocene and Neogene of South China, and probably from North America. *Handeliodendron* is a monotypic genus restricted in southwestern China today^48^ without known fossil record so far.

In order to understand the palaeoenvironment in which these fossil plants inhabited, we compile the environmental niches of the living genera or several genera that are related to the fossils in Supplementary Table 2. The result indicates that most likely the fossil plant assemblage occurred in subtropical to tropical regions at elevation of 800-1200 m assuming the fossil species inhabit a similar niche as their living analogues (Supplementary Table 4). It is also worth noting that among the 15 types of woody dicot leaves in the fossil assemblage, only 5 types have toothed leaf margin, whereas the remaining 10 types all have entire leaf margin (Supplementary Table 2). This appears to indicate high temperature in Nima and Lunpola basin during late Oligocene because of the strong positive relationship between mean annual temperature and the proportion of woody nonmonocot species in a floral sample that has entire leaf margin^56^. However, a quantitatively climatic reconstruction of the fossil assemblage is not possible until more fossil leaves (more than 20 morphotypes) are collected from the Dingqing Formation.

**3. Materials**

Relevant fossil fish material includes seventeen specimens, of which V22782 is the holotype and the specimens V18412-18417, V18581, V18582, V20275, V20276, and V22596-22600 are designated as paratypes. IVPP V18412-18417 are skeletons of varying completeness; V18418 includes some scattered bones of *Eoanabas* preserved on the same slab with a cyprinid dentary (IVPP V 18418.1); V18581 is a complete skeleton; V18582 is a skeleton with the skull and anterior abdominal region disarticulated; V20275 and V20276 are complete skeletons; V20277 is a weathered skeleton; V22596 and 22597 are complete skeletons; V22598 is a partially preserved skeleton with the posterior part of the head well preserved; V22599 is a skeleton with the caudal region missing; V22600 is a complete skeleton. Specimens V18413-18418, V22597, V22598, and V22782 (holotype) are from Jiangnongtangga, V18581, V18582, V20275-20277 and V22596 are from Songwori of the Nima Basin and V22599, V22600 (Supplementary Fig. 5e, f) are from Dayu of Lunpola Basin. Materials for comparison include five cleared and stained skeletons (OP432-436) and one specimen preserved in 95% ethyl alcohol (OP437) of *Anabas testudineus*, two cleared and stained skeletons (OP438-439) and one specimen preserved in 95% ethyl alcohol (OP440) of *Ctenopoma acutirostre*; and one cleared and stained skeleton (OP441) of *Helostoma temminkii.* Fifty specimens of plant megafossils are studied in current paper (IVPP B 2505-B 2554, of which B 2529 and B 2535 are from Nima Basin and the rest are from Lunpola Basin) (Fig. 3; Supplementary Table 2), which were unearthed from the same bed of *Eoanabas*. All specimens are housed at the collections of the Institute of Vertebrate Paleontology and Paleoanthropology (IVPP), Chinese Academy of Sciences, Beijing, China.

1. **Systematic Palaeontology and Supplementary Description**

**Familial Designation** *Eoanabas* has a labyrinth organ and relevant characteristic muscular signatures on the head. These characters indicate its affinity to the anabantoids and their relatives.

Among the lineages previously used as outgroups for assessing the phylogenetic relationships of anabantoids and their relatives, *Badis* and *Nandus* have a unique distally bifurcated hemal spine on the second preural centru^24^, clearly distinguished from the single hemal spine in *Eoanabas*; and their triangular opercle^24^ is radically different from the opercle with notched and spinous posterior margin in the fossil fish herein; additionally, the anal-fin spines are only three in these two taxa^24^, fewer than the anal spines of the fossil fish. Other genera included as the members of family Nandidae by Norris^24^, e.g., *Monocirrhus* and *Polycentrus*, have a notably different opercle and jaw apparatus from those of *Eoanabas*^24^. In a recent phylogenetic analysis, these lineages are found to be only remotely related with anabantoids^57^. Although *Pristolepis* (Pristolepidae) shows some similarities of the opercle with the fossil fish, the construction that its three anal-fin spines are supported by a single, fused pterygiophore^24^ excludes the close relationship of the fossil fish to this taxon.

Channidae (snakehead) is considered to be closely related with the anabantoid fishes^57,58^. The fishes of this family do not have spines in dorsal and anal fins and their opercle is entire in the posterior margin^59^. These features exclude the fossil fish from this family.

Among the anabantoid fishes, Helostomatidae (*Helostoma*) has unique toothless jaws and other extensive specializations in the jaw apparati for filter feeding^24,27^, excluding the possibility of assigning the fossil fish to this family. Members of Osphronemidae have oval or triangular opercle, lacking posterior notch or spines^24,27^, and hence being radically different from that in the fossil fish. Additionally, the osphronemids have either much shorter dorsal fin (e.g., *Betta*, *Trichogaster*) or anal fin with more pterygiophores preceding the first hemal spine (e.g., *Osphronemus* and *Belontia*)^24^ than *Eoanabas*. The pike-head *Luciocephalus* displays a distinct general morphology from that of the fossil fishes, including dorsal and anal fins without spines,. The recently reported fossil gourami from Sumatra, *Ombilincihthys* was placed within Osphronemidae and has different configuration of the opercle and pelvic fin^58^. All considered, we therefore designate the taxonomic affinity of *Eoanabas* to the family Anabantidae.

**Supplementary Description**

The size (total length) of the specimens of *Eoanabas* ranges from ca. 20 mm to 120 mm (Supplementary Table 1). In addition to bearing some characteristic features (e.g., those regarding the opercular structure, see below), *Eoanabas* has a unique mosaic of morphology of its extant Asian and African anabantid relatives.

**Head.** The skull roof of *Eoanabas* lacks any flanges or ridges, and the basic arrangement of the cranial bones is largely similar to that of living anabantids^24^.

**Nasal Region.** The nasal has distinct round lateral curvation (Fig. 1). The lateral ethmoid is crescent and nearly vertically oriented beneath the nasal/frontal conjunction (Fig. 1c, d). In its ventrolateral corner the lateral ethmoid bears a rough area which *in vivo* possibly serves as the attachment point with the lacrimal. In contrast, in living anabantids there is a preorbital process in the comparable position of the lateral ethmoid, linking it with the lacrimal^24^.

**Orbital Region.** The circumorbital bones include a ventrally serrated lacrimal, a small infraorbital 2 and broad infraorbitals 3-5 that cover the cheek completely (Fig. 1 and Supplementray Fig. 5a, b). The lacrimal lacks the prominent anteroventral spike which is common in living anabantids^24^. Posteriorly, the lacrimal articulates posteriorly with the second infraorbital (Figs 1 and 2a) which is relatively small and concaved on the lateral side. Infraorbitals 3 to 5 are expanded to completely enclose the check (Figs 1 and 2a). Similar large infraorbitals 3-5 are only seen in *Anabas*^24^; however, the sensory pores open between these elements, a character known only in African anabantids^24^. The suborbital shelves of the infraorbitals 3-5 are developed. The dermosphenotic is small and largely triangular in shape, lying anterior to the sphenotic (Fig. 1).

**Otic and Occipital Regions.** The sphenotic separates the dermosphenotic from the pterotic and contacts anteromedially with the frontal (Fig. 1 and Supplementary Fig. 5a, b). The sphenotic does not bear a postorbital process, as in African *C. multispine* species assemblage, most species of *C. petherici* and *Sandelia bainsii*^24^. Just behind the sphenotic/pterotic junction, the pterotic bears a sensory canal opening in addition to a posterior sensory canal opening (Fig. 1 and Supplementary Fig. 5a, b), which is present in *Anabas* but not in African forms^24^. The pterotic bears in the posterolateral corner a depressed triangular facet for the insertion of the levator operculi muscle as in living anabantids^24^ (areas in red color in Fig. 1d and Supplementary Fig. 5b). And relative to the length of the pterotic, the muscular facet for the levator operculi muscle on the bone is proportionally longer than that in most of living anabantids^24^. Right beneath this muscular facet is the suprabranchial chamber (Fig. 1c, d) housing the labyrinth organ referred below. The parietal is flat and roughly quadrate in shape with a relatively long parietal/pterotic suture (Fig. 1 and Supplementary Fig. 5a, b). The supraoccipital is somehow acute at the anterior end, lacking a supraoccipital crest. The extrascapular is plate-like (Supplementary Fig. 5a, b). The parasphenoid shaft is straight without oral or orbital process (Supplementary Figs 3a, b, g, 4k−m).

**Suspensorium and Jaw Apparati.** The hyomandibular was preserved with only the anterior flange and dorsal part of the vertical axis exposed in specimen IVPP V18412 (Fig. 1c, d). Compared to that in most living anabantids^24^, the anterior flange is rather reduced and recedes rapidly towards the vertical axis such that the anteroventral margin dose not continue to the base of the vertical hyomandibular shaft, nor is it expansive enough to contact with the metapterygoid. The pointed process on the hyomandibular underlying the preopercular is most likely not developed. The quadrate was preserved with the articular condyle clearly observable, but the structure of its preopercular process cannot be determined. The preopercular is “L”-shaped with the horizontal arm being shorter than the vertical one, and its posteroventral corner is not serrated (Fig. 1c, d and Supplementary Figs 3a, b and 5a, b).

The dentary has a V-shaped recess in its posterior margin for the insertion of the anguloarticular (Fig. 1a, b and Supplementary Fig. 4j, k). Along the anterior oral edge of the dentary are some stout and slightly recurved teeth (Fig. 1a, b and Supplementary Fig. 3f), whereas in the posterodorsal corner is the deep and obtuse ascending process (Fig. 1a, b and Supplementary Fig. 4j, k) as in living anabantids^24^. On lateral side of this bone, three openings of the mandibular sensory canal are observable (Fig. 1a, b). The anguloarticular bears a broad but shallow coronoid process (Fig. 1a, b and Supplementary Fig. 3j, k). In the upper jaw, the premaxilla has an alveolar process nearly at a right angle and similar in length to its toothed arm (Figs 1a, b and 2a, b, Supplementary Fig. 3a, b, 4k−m). The premaxillary articular process is developed. No postmaxillary process is present. The maxilla is relatively short and stout with a clubbed posterior end (Figs 1 and 2a, b, Supplementary Fig. 4k), which is of a similar appearance as that in African anabantids except *Ctenopoma petherici* clade^24^.

**Opercular Region.** A V-shaped strut on the inner side emerges from the articular socket with the hyomandibular (Fig. 1a, b and Supplementary Figs 3a, b, 4k, 5a, b) and terminates from the spines bounding the posterior opercular notch. The subopercle and the interopercle are not serrated in the distal edge. Six branchiostegal rays are developed with only the posterior-most one attached to the posterior ceratohyal (Supplementary Fig. 4l, m). The urohyal does not bear a horizontal process in the posterodorsal edge (Supplementary Fig. 4l, m).

**Branchial Skeleton.** Relics of the labyrinth organ is preserved in specimen V19581a (Fig. 2c, d). This structure is reminiscent of the characteristic appearance of the expanded lamellae of the accessory air-breathing organ (i.e., labyrinth organ, the extensively modified first epibranchial) in living anabantids^24^ (Fig. 2e, f), and suggests that the fossil taxon should also have such an organ (Fig. 2h). Although the relevant fossil individual (V19581a) is relatively small in body length (ca. 2 cm) (Fig. 2c), that organ should have already been in place given that the development of the labyrinth organ in living anabantid (*Anabas*) begins as early as before the fish reaches 1.5 cm long^60^. The fifth ceratobranchial carries numerous teeth (Supplementary Fig. 3h), biting against those of the pharyngobranchial 3 and/or 4 (Supplementary Fig. 3h). Impressions of the gill rakers of the first ceratobranchial are preserved in the holotype (Fig. 1a, b), whereas gill rakers of other gills arches are not detectable.

**Pectoral and Pelvic Girdle.** The ventral process of the posttemporal is relatively long and narrow. The cleithrum has a pointed dorsal process (Fig. 1c, d and Supplementary Figs 4l and 5a, b). The dorsal postcleithrum is ovoid in shape (Supplementary Fig. 4l, m). The stout descending process of the ventral postcleithrum extends downward to contact the lateral process of the pelvic plate (Supplementary Figs 3g and 4l, m). The pelvic plate is roughly triangular in shape and lying flat (Fig. 1 and Supplementary Fig. 4l, m) as in *Anabas*^24^ but different from the dorsally angled one in African forms^24^. The pelvic fin has a spine anteceding five unmodified fin rays (Fig. 1a, b and Supplementary Figs 3g, 4a, b, l, m).

**Axial Skeleton.** The vertebral column is largely straight except in the most anterior length where a slightly dorsal deflection occurs (Fig. 1a, b and Supplementary Figs 3a, b, 4j−m), comprising 24-25 vertebrae (including the ultimate compound vertebra) (Supplementary Table 1). In the abdominal region, the anterior neural spines closely approach the posterior face of the dorsal fin pterygiorphores as in African anabantids except *C. multispine* clade^24^. According to definition of Patterson & Johnson^61^ and Gemballa & Britz^62^, the intermuscular bones are divided into two types: the epineurals and the epipleurals (Fig. 1a, b and Supplementary Fig. 3a,b).

**Unpaired Fins and Support.** The truncated caudal fin has a principal fin-ray formula I-7/7-I. The internal supporting skeleton of the caudal fin involves three vertebrae, one uroneural, two epurals, one parhypural, and five hypurals. The parhypural is similar to or slightly narrower than the hypurals 1 and 2 in width and thus proportionally much narrower than that in living anabantids^24^. The hypural 4 and 5 are likely fused to the compound vertebra. And the hypural 5 is much slender than the hypural 4. The uronerual is long, reaching as far as the distal end of the hypural 5.

The dorsal and anal fins are long-based with the fin formula of D. XIII-XIV/7-8, and A. VI-IX/7-9, respectively (Supplementary Table 1). The number of anal fin spines is relatively low compared to that of living anabantids except a few species of *Microctenopoma*, e.g., *M. milleri*, which has a fin formula of D. XIV/9-10, A. VI-VII/10-11)^63^. The posterior most dorsal fin pterygiophores is singular as in African anabantids, unlike the divided ones in *Anabas*^24^. The first anal fin pterygiophore carries two spines (Fig. 1a, b and Supplementary Figs 3a, b, j, k, 4k−m). The posterior most dorsal and anal fin pterygiophores are singular. Three supraneurals are developed (Fig. 1a, b and Supplementary Figs 3h, 4j, l, m). The dorsal and anal fin rays do not show any modification.

**Scales.** The head is covered on the top and in the cheek region (except the supposed males described below) by cycloid scales (Fig. 1c, d and Supplementary Figs 4l, m, 5a), whereas the trunk is covered by ctenoid scales (Supplementary Figs 3c, e).

**5. Phylogenetic Analyses**

This late Oligocene anabantid from Tibet, *Eoanabas,* evidently shows a diagnostic character combination of the family Anabantidae (see Systematic Palaeontology section above). Notably, it possesses a mosaic of anabantid features, some of which resemble Asian relatives and others resemble certain African members of the family (see Systematic Palaeontology section above for details). To resolve its phylogenetic position within the group, we conducted a morphology-based phylogenetic analysis, and as a result, the fossil taxon was placed as the most basal lineage, with all living Asian and African anabantids as its anabantid relatives (Supplementary Fig. 6), of which the African taxa are monophyletic and sister to the Asian genus *Anabas*. It is notable that our phylogenetic analysis yielded higher resolution for the living anabantid interrelationships (Supplementary Fig. 6) than a previous comprehensive morphology-based analysis^24^. Moreover, the result is compatible with that of a molecular-based research^25^ and the backbone-constrained analysis in the monophyly of African lineages and the inclusion of the fossil taxon within the family Anabantidae in the latter analysis, despite some topological differences regarding the interrelationships of the African taxa (Supplementary Fig. 9). These uncertainties reflect in part the morphology/molecular conflict (it is, however, beyond the scope of the current study to resolve this conflict) and also suggest that future morphology- and molecular-based studies are needed to more rigorously test the anabantid intrarelationships as claimed in the molecular study^25^.

The systematic scheme and nomenclature of living anabantids follow Norris^24,64^ and Tim^23^.

**6. Supplementary Tables**

**Supplementary Table 1 | Meristic characters of *Eoanabas thibetana* gen. et. sp. nov. from the late Oligocene of Tibet, China.**

|  | TL | SL | HL | HL/SL | DF | AF | CF | AMV | CV |
| --- | --- | --- | --- | --- | --- | --- | --- | --- | --- |
| V 18412 | ～117 | 94 | 37 | 45.1% | XIII-XIV/8 | >V/9 | I-7/7-I | ？ | ？ |
| V 18413 | ～40 | ? | ? | ? | XIII/8 | VII/8 | I-7/7-I | ？ | ? |
| **＊V 18414** | 65 | 53 | 22 | 41.5% | XIV/>5 | IX/>8 | ? | ？ | ? |
| V 18415 | ？ | ？ | ？ | ？ | XIV/? | ?/8 | ？ | ？ | ? |
| V 18416 | ？ | ？ | ？ | ？ | >XI/7 | ?/≥7 | I-7/7-I | ？ | ？ |
| V 18417 | ？ | ？ | ？ | ？ | XIV/? | IX/? | ？ | ？ | ？ |
| V 18581 | 22 | 18.5 | 8.5 | 45.94% | XIV/7 | ?VII/8 | ? | 10 | 15 |
| **＊V 18582** | ？ | ？ | ？ | ？ | >XI/8 | VIII/8 | I-7/7-I | ? | 15 |
| V 20275 | ~50 | ？ | ？ | ？ | XII/7 | VI/7 | I-7/7-I | 10 | 14 |
| V 20276 | 23 | 19 | 8 | 42.11% | XIV/8 | VII/9 | I-7/7-I | 10 | 14 |
| V 22596 | 28 | 23.5 | ? | ? | XIII/7 | VI/? | ? | ？ | ？ |
| **＊V 22597** | ～70 | ? | ? | ? | ? | IX/? | ? | ？ | ？ |
| V 22599 | ？ | ? | 14 | ? | XIV/8~9 | VIII/6~8 | ? | ？ | ？ |
| V 22600 | 90 | 75 | 34 | 45.33% | XIV/9 | VII/9 | I-7/7-I | ？ | ？ |
| V 22782 | 55 | 46 | 19 | 41.3% | XIII/8 | VII/8 | I-7/7-I | 11 | 14 |

Abbreviations: **AF**, anal fin formula. **AMV**, number of abdominal vertebrae. **CF**, caudal fin formula. **CV**, number of caudal vertebrae. **DF**, dorsal fin formula. **HL**, head length including opercle. **SL**, standard length (measured from rostral tip to caudal fin base). Specimens with asterisks (*) represent supposed males. Unit of measurements: mm.

**Supplementary Table 2 | Plant megafossil assemblage coexisting with *Eoanabas* from the late Oligocene Dingqing (DQ) Formation of Lunpola and Nima basins. Definition of leaf margin and leaf size accords to ref. 65**.

| Non-monocot Angiosperm Leaves | | | | |
| --- | --- | --- | --- | --- |
| Morphotype | Leaf Margin | Leaf Size | Affinity | Remark |
| DQ01 | Entire | Microphyll | *Pistacia*, Anacardiaceae | IVPP B 2508  IVPP B 2509 |
| DQ02 | Entire | Notophyll | Hippocastanaceae | IVPP B 2513 |
| DQ03 | Entire | Mesophyll | *Exbucklandia*, Hamamelidaceae | IVPP B 2516 |
| DQ04 | Toothed | Microphyll | Araliaceae | IVPP B 2515 |
| DQ05 | Entire | Microphyll | Bignoniaceae? | IVPP B 2517 |
| DQ06 | Entire | Microphyll | indet dicot | IVPP B 2518 |
| DQ07 | Toothed | Microphyll | indet dicot | IVPP B 2519 |
| DQ08 | Entire | ? | indet dicot | IVPP B 2520 |
| DQ09 | Toothed | ? | Fagales | IVPP B 2521 |
| DQ10 | Entire | Microphyll | Lauraceae? | IVPP B 2522 |
| DQ11 | Toothed | Microphyll | *Populus*, Salicaceae | IVPP B 2523 |
| DQ12 | Entire | Microphyll | indet dicot | IVPP B 2524 |
| DQ13 | Entire | Microphyll | indet dicot | IVPP B 2525 |
| DQ14 | Entire | Mesophyll | Magnoliales | IVPP B 2526 |
| DQ15 | Toothed | Microphyll | indet dicot | IVPP B 2527 |
| DQ16 | ? | ? | *Limnobiophyllum*, Lemnoideae, Araceae | IVPP B 2514 |
| Monocot Leaves | | | | |
| DQ17 |  |  | Arecaceae | IVPP B 2505 |
| DQ18 |  |  | Araceae? | IVPP B 2528 |
| DQ19 |  |  | *Typha*, Typhaceae | IVPP B 2529 |
| DQ20 |  |  | indet monocot | IVPP B 2530 |
| DQ21 |  |  | indet monocot | IVPP B 2531 |
| DQ22 |  |  | indet monocot | IVPP B 2532 |
| DQ23 |  |  | indet monocot | IVPP B 2533-2534 |
| Fruits & Seeds | | | | |
| DQ24 |  |  | *Koelreuteria*, Sapindaceae | IVPP B 2506  IVPP B 2507 |
| DQ25 |  |  | *Cedrelospermum*, Ulmaceae | IVPP B 2510-2512 |
| DQ26 |  |  | Araceae? | IVPP B 2535 |
| DQ27 |  |  | indet fruit | IVPP B 2536 |
| DQ28 |  |  | indet fruit | IVPP B 2537 |

**Supplementary Table 3 | Distribution range, climatic type and temperature of the habitat of extant anabantids and outgroup taxa included in the phylogenetic analyses. Data from** [**www.fishbase.org**](http://www.fishbase.org/) **and ref. 66**.

| Taxa | | Distribution | Climate, Temperature |
| --- | --- | --- | --- |
| Badidae | *Badis* | Pakistan, India, Nepal, Bangladesh, and Burma | Tropical; 14℃-30℃ |
| Helsotomatidae | *Helostoma temmincki* | central Thailand, the Malay Peninsula, Sumatra, Java, and Borneo | Tropical; 22℃-28℃; |
| Anabantidae | *Anabas* | Pakistan, India, Bangladesh, Sri Lanka, Burma, the Malay Peninsula, Indonesia, Borneo, Philippines | Tropical; 22℃-30℃; |
|  | *Ctenopoma* | Western and central Africa, mainly along the Congo (Zaire), the Zambezi river system | Tropical; 20℃-30℃ |
|  | *Microctenopoma* | primarily Zairian | Tropical; 18℃-30℃ |
|  | *Sandelia* | Eastern and southern Cape coastal rivers in South Africa | Temperate; 18℃-22℃ |

**Supplementary Table 4 | Plant megafossils coexisting with *Eoanabas* from the late Oligocene Dingqing Formation of Lunpola basins, and the habitats of their living analogues based on Flora of China^46−51^.**

| Fossil taxon | Living analogue | Elevation | Habitat |
| --- | --- | --- | --- |
| Arecaceae^46^ | Arecaceae genera with palmate leaves | <2500 m | Subtropical and tropical regions |
| Araliaceae^47^ |  | 0–3500 m | Warm temperate, subtropical and tropical regions |
| *Koelreuteria*^48^ | *K. bipinnata*-type | 100–2600 m | Subtropical and tropical regions |
| *Handeliodendron*^49^ | *Handeliodendron* | 500–1200 m | Subtropical regions |
| *Pistacia*^50^ | *Pistacia* | 100–3600 m | Warm temperate, subtropical and tropical regions |
| *Exbucklandia*^51^ | *Exbucklandia* | 800–1500 m | Subtropical and tropical regions |

**7. Supplementary Figures and Captions**

**
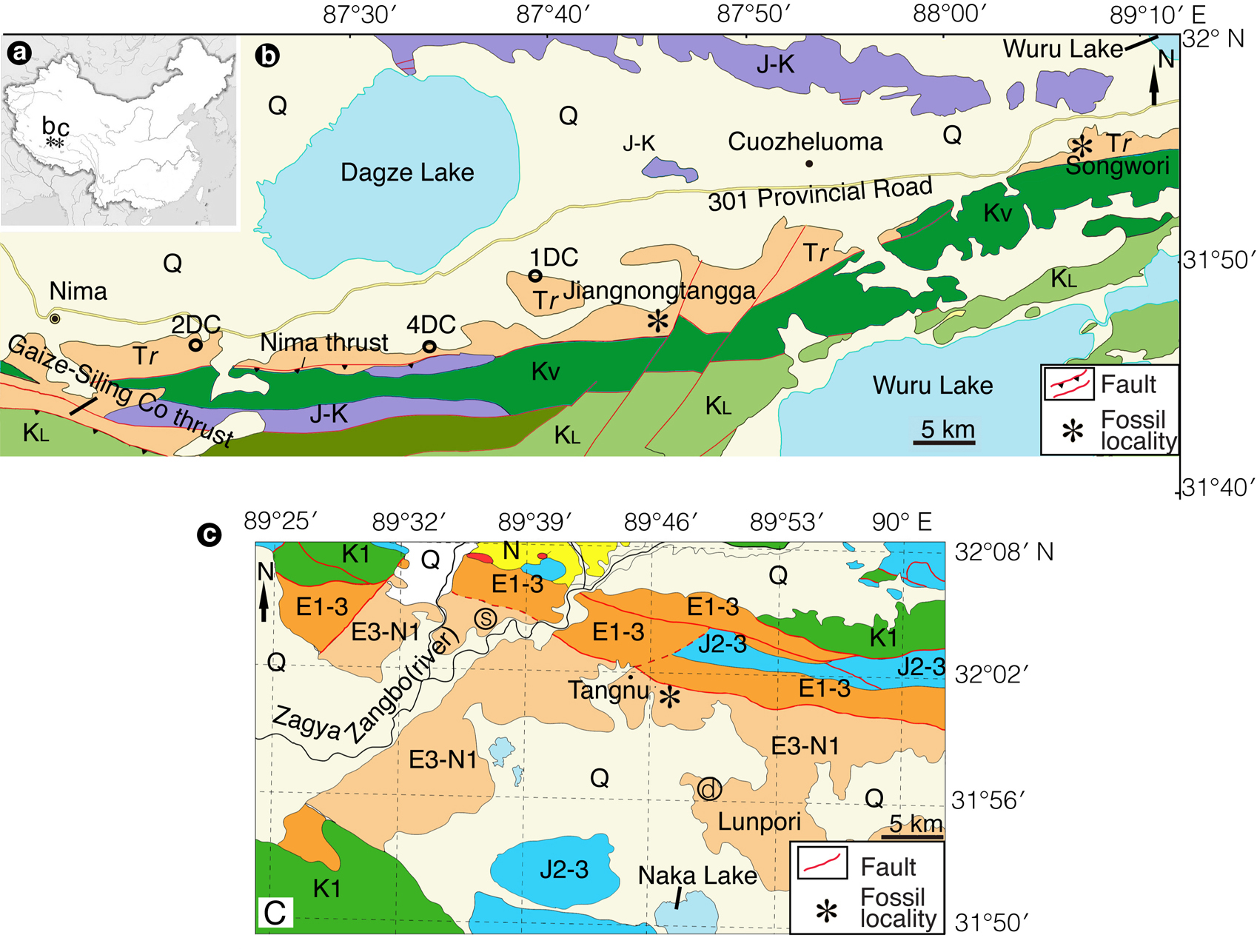
**

**Supplementary Figure 1 | Location of the Nima and Lunpola basins and the simplified general geological settings of the fossil localities. (a**) Location of fossil sites in the map of China’s mainland as marked by the asterisks. (**b**) Simplified geological map of the south margin of Nima Basin showing the position of the fossil localities marked by the asterisks, with location I (Jiangnongtangga section) yielding the holotype (V22782), V18412-18418, and location II (Songwori section) yielding V 18581, 18582, 20275-7. 1DC, 2DC and 4DC are the positions of the sampled sections investigated in refs. 6, 40, 41. (**c**) Simplified geological map of the Lunpola Basin showing the position of our fossil locality marked by the asterisk; other fossil localities refer to the sampled sections in ref. 20 and ref. 26, labeled with D and S in black circles, respectively. The maps are created by authors using “Adobe Illustrator CS3” software. URL <http://www.adobe.com/>. Geological data are adopted from refs. 39, 42−44. Abbreviations: E1-3, Eocene-Oligocene Niubao Formation; E3-N1, Oligocene-Miocene Dingqing Fm.; J1-2, Lower-Middle Jurassic Mugagangri Fm.; J2-3, Middle-Upper Jurassic Jienu Group; J-K, Jurassic-Cretaceous shales, siltstone, turbiditic sandstone, metasedimentary-matrix mélange; K1, Lower Cretaceous Guiyayan Fm. or Qulashen Fm.; Kcl, Cenomanian conglomerate with mainly Aptian-Albian limestone clasts; KL, Aptian-Albian massive limestone; Kv, Albian volcanic flows, tuffs, breccias; volcanic sandstone and conglomerate; Q, Quaternary; T*r*, Tertiary red beds of the South Nima area, corresponding to the Dingqing Formation in the Lunpola Basin^41,42^.


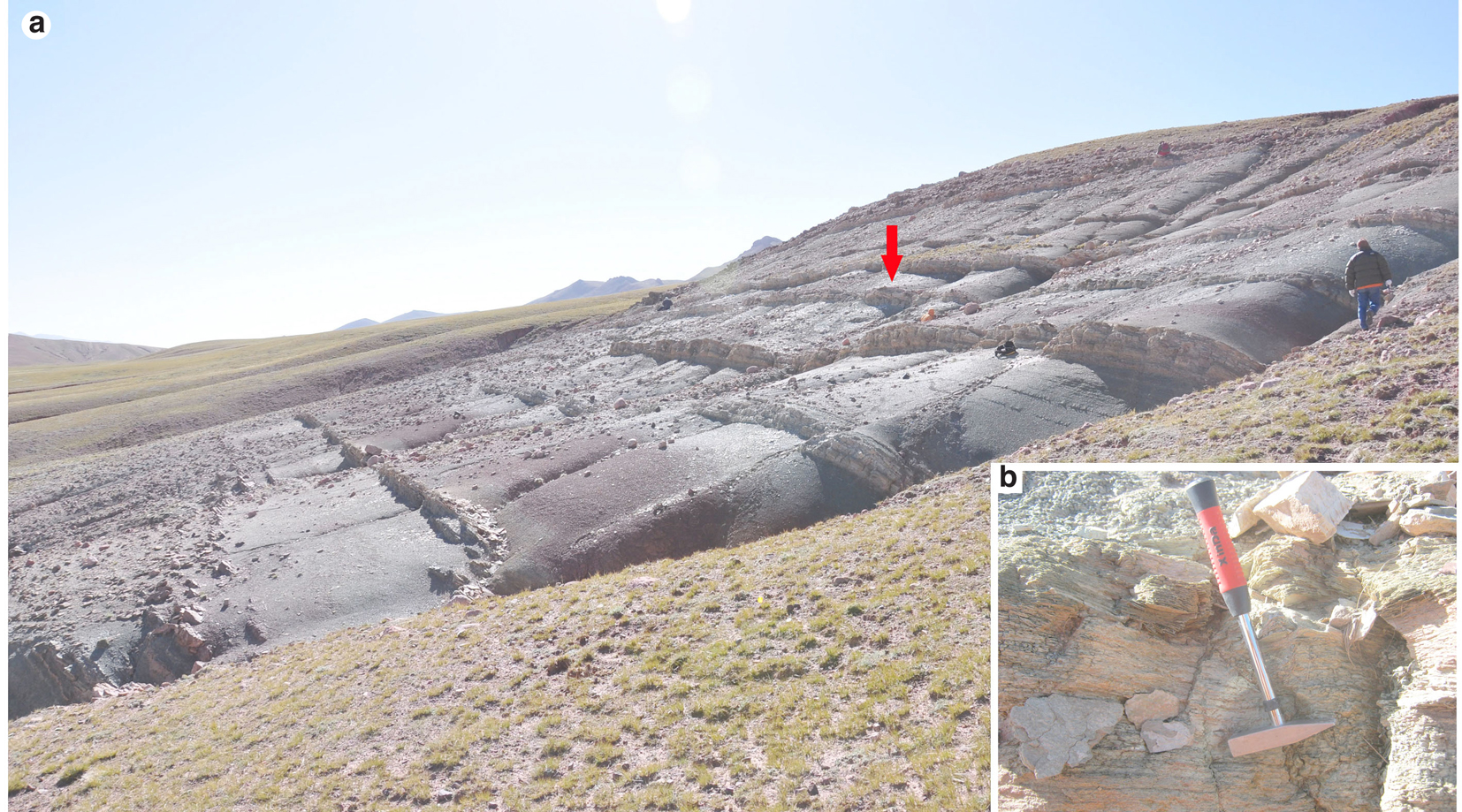


**Supplementary Figure 2 |** Exposures of Jiangnongtangga section (Type locality) where the holotype (V22782) of *Eoanabas thibetana* was found, Nima Basin, Tibet, China. (**a**) Photograph of the fossiliferous strata, red arrow pointing the layer where the holotype was unearthed, the photographer facing east, and the author on the right for scale. (**b**) Close-up of the fish-bearing rocks, hammer is ca. 30 cm long.


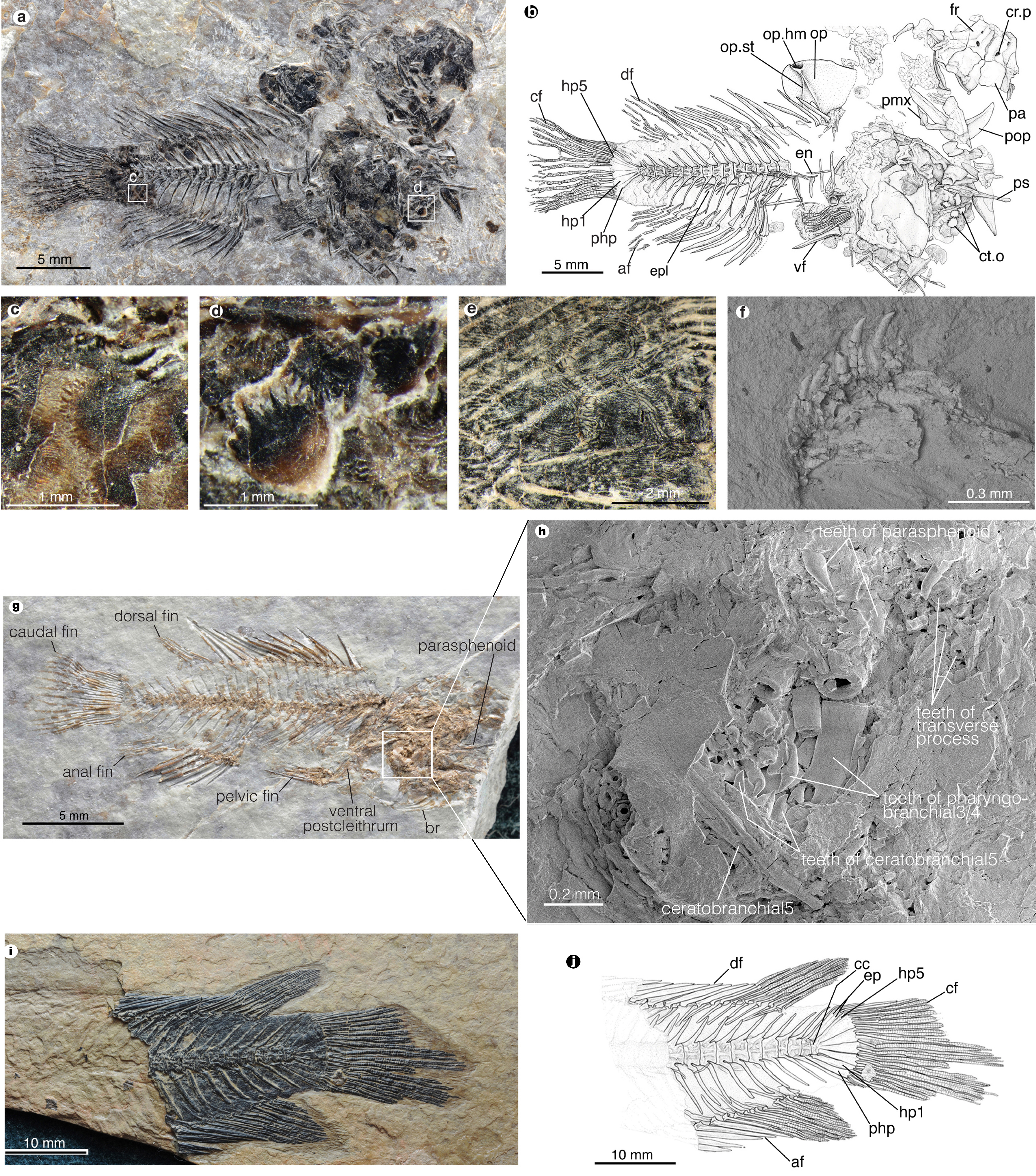


**Supplementary Figure 3 | *Eoanabas thibetana* gen. et sp. nov.** **from the upper Oligocene of central Tibet.** (**a**) Photograph and (**b**) Line drawing of IVPP V18582. (**c**) Photograph of ctenoid scales on caudal peduncle of IVPP V18582 (**a**). (**d**) Photograph of postocular contact organ of IVPP V18582 (**a**). (**e**) Photograph of ctenoid scales on caudal peduncle of IVPP V18412. (**f**) Teeth on the dentary of IVPP V18581b. (**g**) Photograph of IVPP V18581a. (**h**) Scanning Electron Microscope (SEM) images of branchial region in (**g**). (**i**) Photograph and (**j**) Line drawing of the trunk and tail of IVPP V18414a. Abbreviations: af, anal fin; cc, compound centrum; cf, caudal fin; cr.p, coronal pore; ct.o, contact organ; df, dorsal fin; ep, epural; epl, epipleural; fr, frontal; hp1, hypural1; hp5, hypural5; op, opercle; op.hm, opercular articular socket to hyomandibular; op.st, V-shaped strut on inner side of opercle; php, parhypural; pop, preopercle; r, rib; vf, pelvic fin. For other abbreviations see the figure captions in main paper.

**
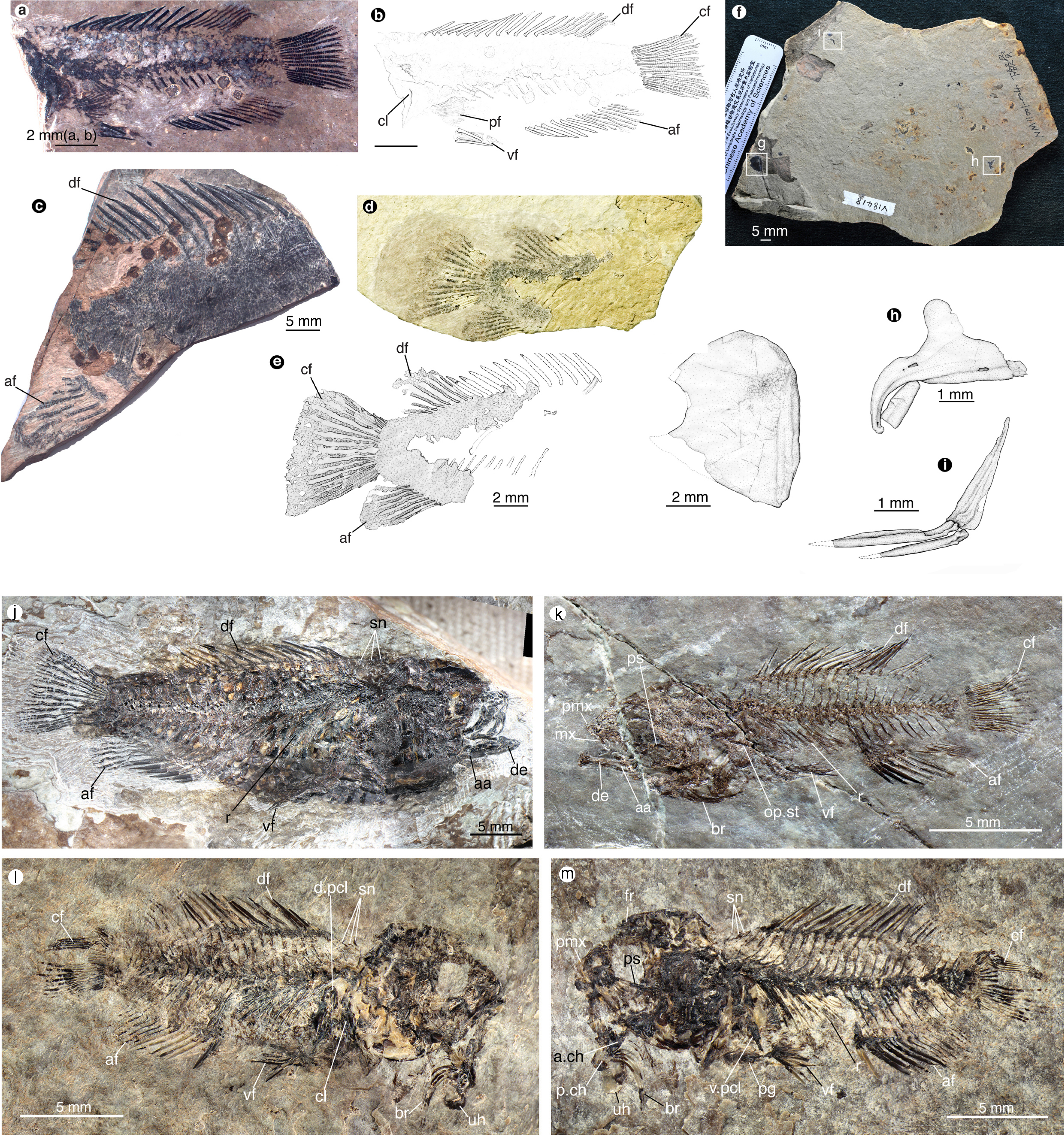
**

**Supplementary Figure 4 | *Eoanabas thibetana* gen. et sp. nov. from the upper Oligocene of central Tibet.** (**a**) Photograph of IVPP V18413a. (**b**) Line drawing of A. (**c**) Photograph of IVPP V18415. (**d**) Photograph of IVPP V18416. (**e**) Line drawing of (**d**). (**f**) Photograph of a slab with some relics of *Eoanabas* (IVPP V18418) and other coeval fish (IVPP V 18418.1). (**g**) Line drawing of an opercle of *Eoanabas thibetana*. (**h**) Line drawing of dentary of an undetermined cyprinid fish (IVPP V 18418.1). (**i**) Line drawing of first anal pterygiophore and the associated spines (IVPP V18418). (**j**) Photograph of IVPP V20275a. (**k**) Photograph of IVPP V18581b. (**l**) Photograph of IVPP V20276a. (**m**) Photograph of IVPP V20276b. Abbreviations: aa, anguloarticular; a.ch, anterior ceratohyal; cl, cleithrum; de, dentary; d.pcl, dorsal postcleithrum; p.ch, posterior ceratohyal; pf, pectoral fin; pg, pelvic plate; uh, urohyal. For other abbreviations see the caption of Supplementary Figure 3 above.

**
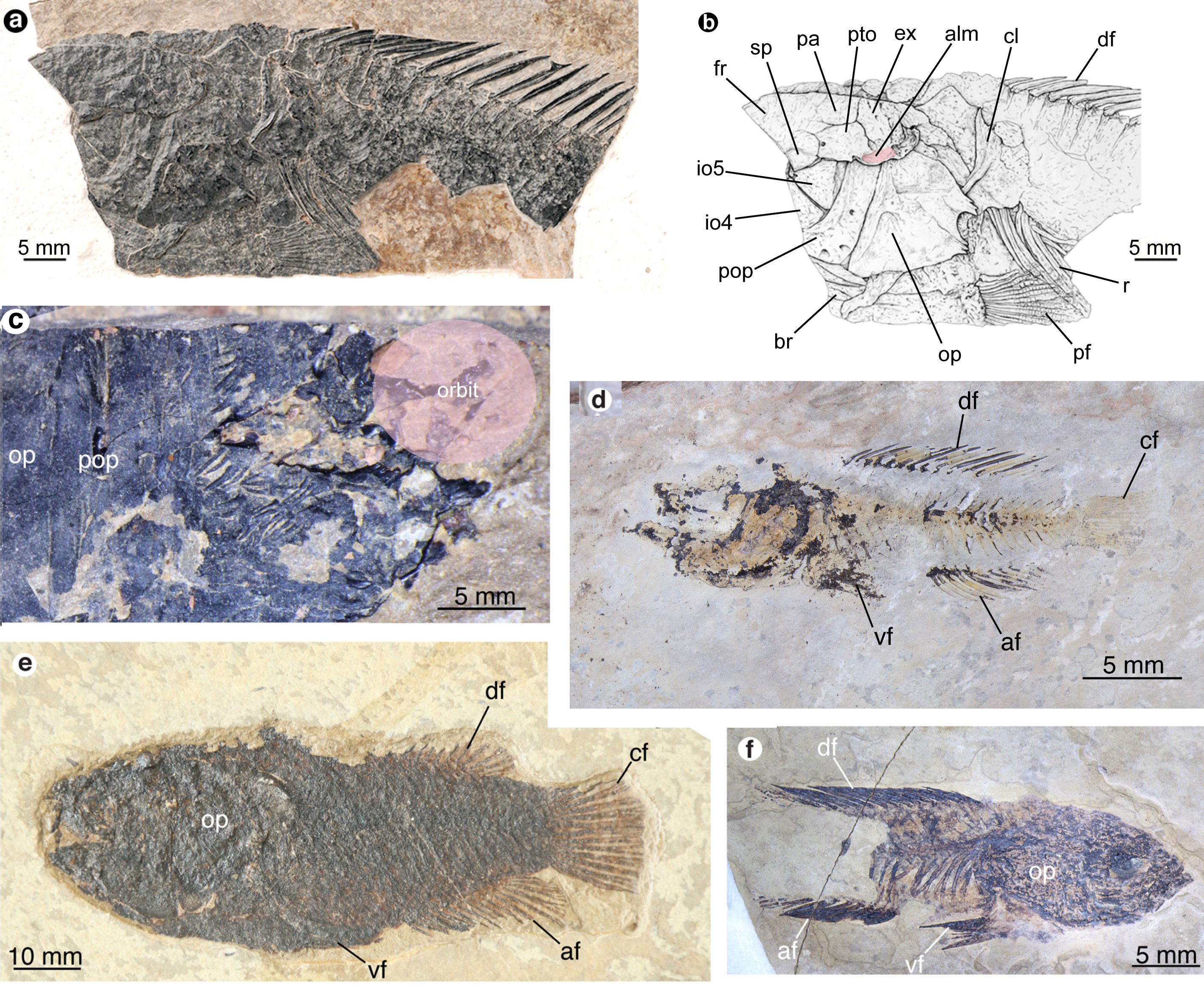
**

**Supplementary Figure 5 | *Eoanabas thibetana* gen. et sp. nov.**

(**a**) Photograph of IVPP V22598. (**b**) Close-up of the postocular contact organ in IVPP V22597a. (**c**) Photograph of IVPP V22596a. (**d**) Photograph of IVPP V22596b. (**e**) Photograph of IVPP V22600. (**f**) Photograph of IVPP V22599a. Abbreviations: br, branchiostegal rays; ex, extrascapular; io4, infraorbital4; io5, infraorbital5; pto, pterotic; r, rib; sp, sphenotic. See other abbreviations in main text figures and supplementary figures 3 and 4.


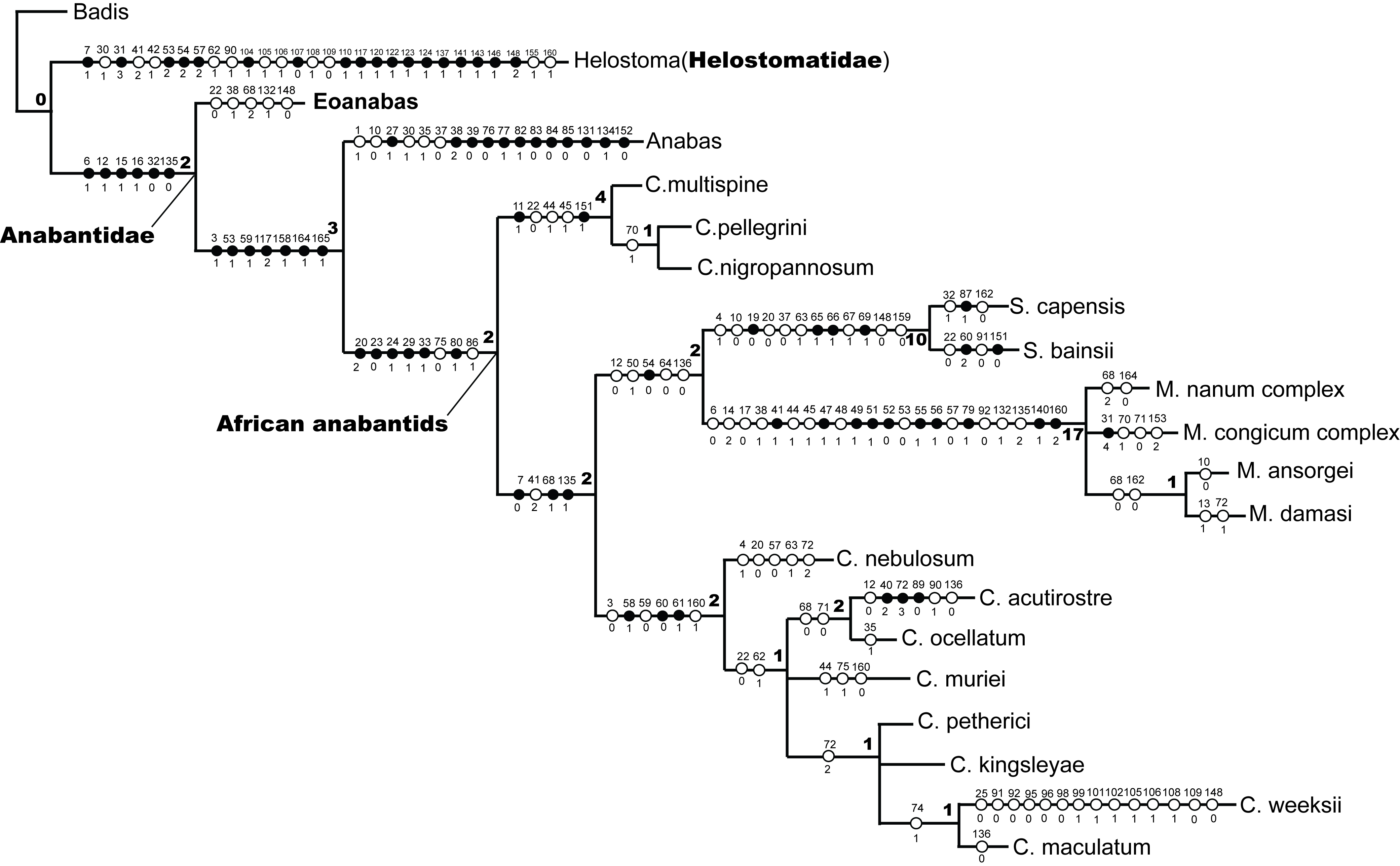


**Supplementary Figure 6 | Phylogenetic position of *Eoanabas thibetana* gen. et sp. nov.** **within the Anabantidae.** A strict consensus chronogram of 2 most parsimonious trees. ACCTRAN optimization of characters is given for each node on the lines with

character number above and character states below the lines. Characters above black circles are the ones with consistency index of 1. Tree length: 315; Consistency Index (CI): 0.641; Retention Index (RI): 0.733. Abbreviations: C., *Ctenopoma*; com., species complex; M., *Microctenopoma*; S., *Sandelia*.

**
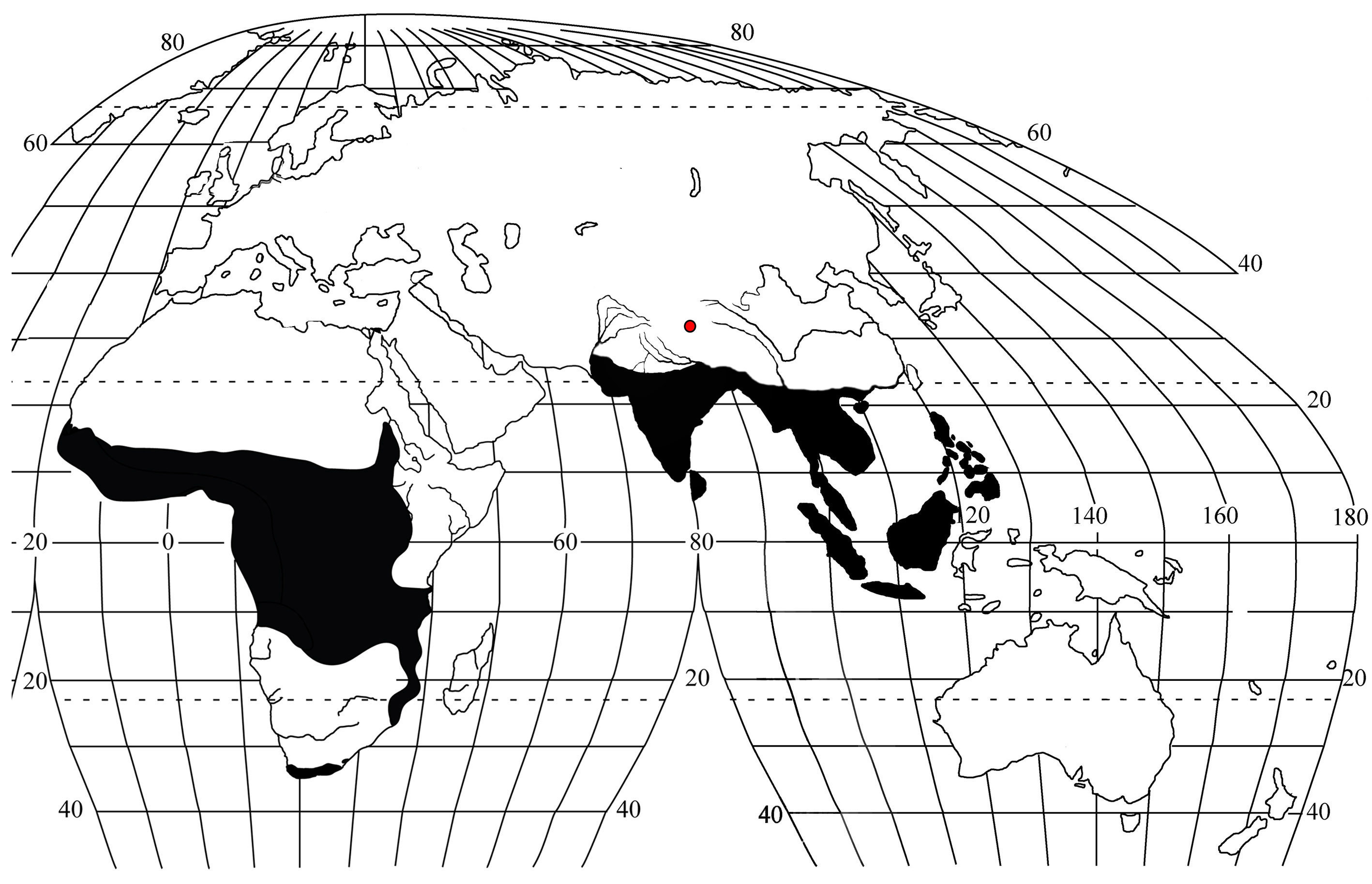
**

**Supplementary Figure 7 | Distribution of extant anabantid fishes with the red spot marking the location of fossil anabantid *Eoanabas thibetana* gen. et sp. nov.** Distribution range after refs. 23 and 66, and the anabantid distribution in China was partially revised (climbing perches do not occur in Taiwan, Fujian and northern Guangdong of China) based on data in ref. 67 and personal communications with Bin Kang (2014). The images are created by authors using “Adobe Illustrator CS3” software. URL <http://www.adobe.com/>.

**
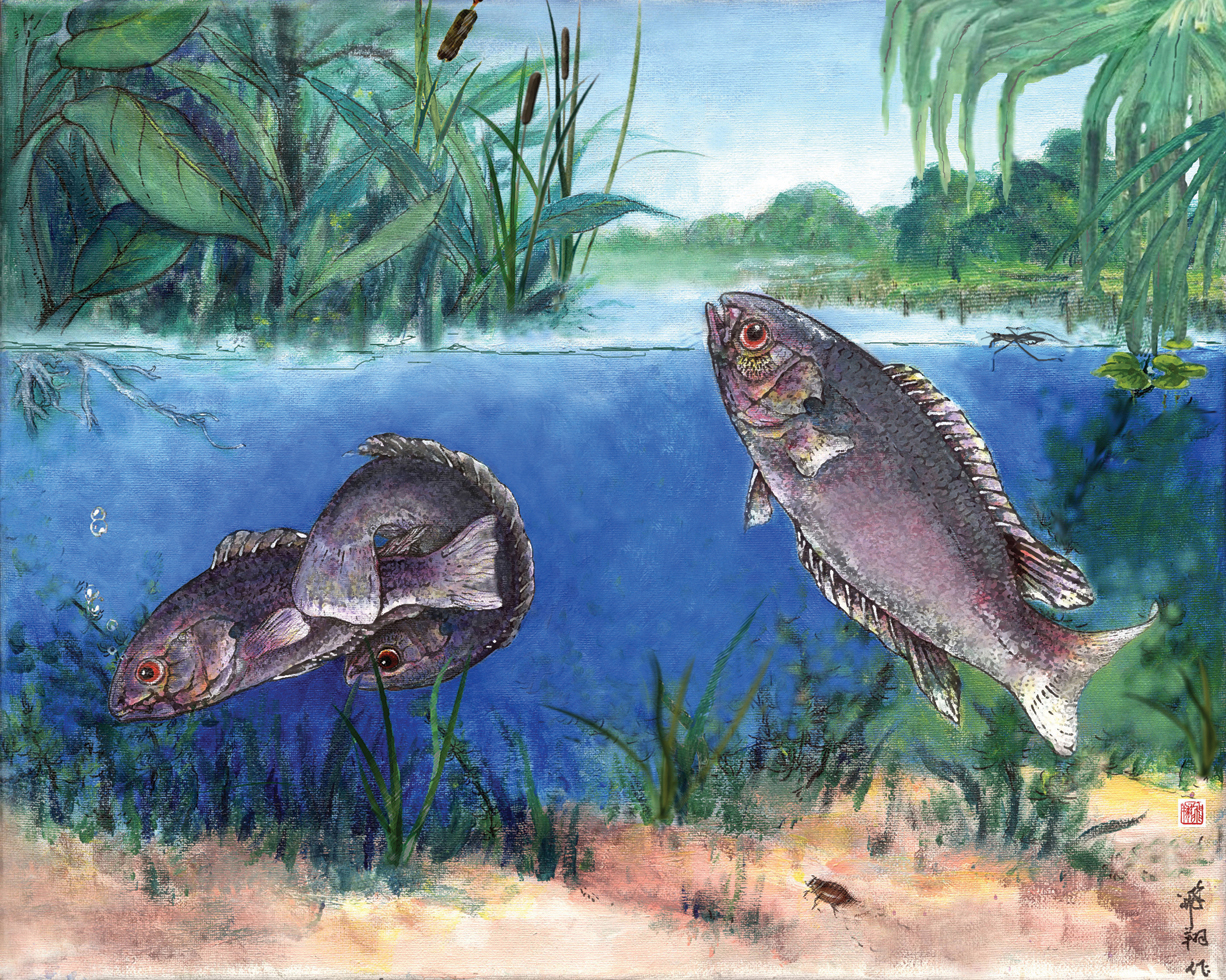
**

**Supplementary Figure 8 | Life restoration of *Eoanabas thibetana* gen. et sp. nov.** Life under a warm, humid, and well-vegetated environment in central Tibet ca. 26 million years ago, showing its mating clasp (left) and surfacing to gulp air for accessary air breathing (right). The drawing was produced by Feixiang WU.

**
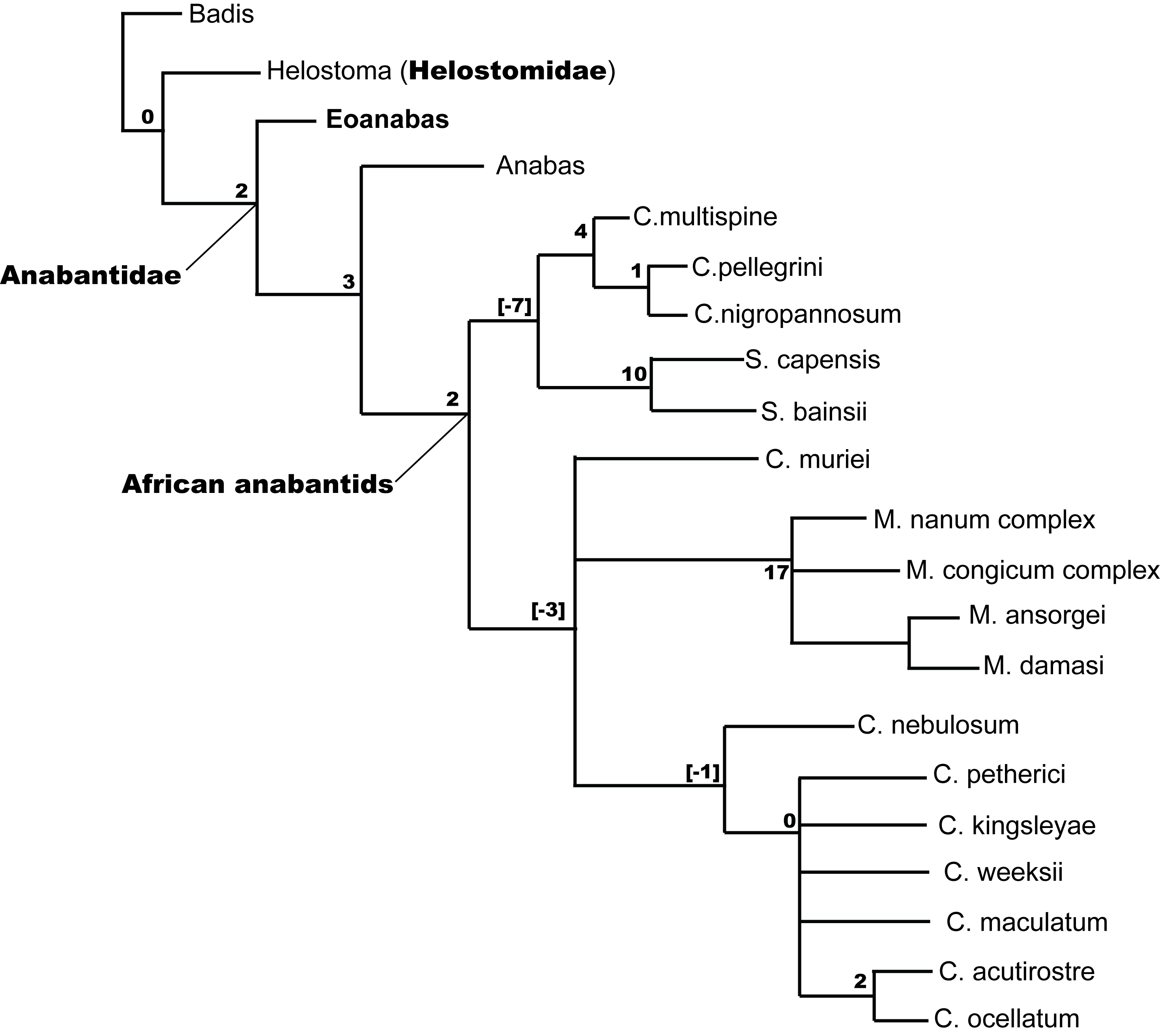
**

**Supplementary Figure 9** | **Backbone-constrained phylogenetic analysis for testing systematic position of *Eoanabas*.** Strict consensus of 10 most parsimonious trees of backbone-constrained analysis. Tree length: 332; Consistency Index (CI): 0.608; Retention Index (RI): 0.693. Numerical values below nodes denote Bremer decay indices. Abbreviations: **C.**, *Ctenopoma*. **com.**, species complex. **M.**, *Microctenopoma*. **S.**, *Sandelia*.

**8. Appendix**

***Appendix 1***

Characters Description: (N: Norris^24^, numbering of charecters 1-131 consistent with those in ref. 24)

1. Myodomal cup: (0) absent; (1) attached to ventral plate of mesethmoid; (2) no cup but having detached anterior myodone bone. (according to ref. 24)

2. Nasals conjoined medially: (0) no; (1) yes.

3. Articulation of second infraorbital with lachrymal: (0) posterad; (1) posterodorsad;

(2) not at all. (from N3, but designating 0 for ‘posterad’ and 1 for ‘posterodorsad’ based on Norris’ description^24^)

4. Lateral faces of sphenotics covered by pterotics: (0) no; (1) yes.

5. Exoccipitals completely bound foramen magnum: (0) no; (1) yes.

6. Supraoccipital crest: (0) absent; (1) present.

7. Epioccipital process: (0) short; (1) long; (2) absent.

8. Parasphenoid shaft: (0) straight; (1) straight, but narrowed at ascending wings. (from N8)

9. Transverse process of parasphenoid: (0) absent; (1) present.

10. Transverse process of parasphenoid: (0) short; (1) long; (2) no process.

11. Terminal process of transverse process: (0) absent; (1) present; (2) no process.

12. Extrascapulars: (0) tubular; (1) plate-like.

13. Anterior flanges of hyomandibular articulate with metapterygoids: (0) no; (1) yes.

14. Postmaxillary processes of premaxillary: (0) high; (1) low; (2) none. (from N8)

15. Support struts on medial surface of operculars: (0) single; (1) V-shaped; (2) no struts.

16. Posterior notch on operclars: (0) absent; (1) present.

17. Stout ligament joining paired fifth ceratobranchials: (0) absent; (1) present.

18. Form of valvular organ: (0) multiple flat elements on ceratobranchial 1 and 2; (1) cupped structure; (2) double cup; (3) multiple flat elements on ceratobranchial 1 only; (4) no valvular organ.

19. Pharyngobranchial 2 in cross-section: (0) ovoid; (1) round; (2) plate.

20. Valvular organ: (0) none; (1) thin plates; (2) bowl-shaped.

21. Postocular contact organs: (0) absent; (1) present.

22. Postorbital process of sphenotic: (0) absent; (1) present.

23. CLL (Cranial Lateralis Line) pore at anterior border of pterotic: (0) absent; (1) present.

24. Median descending process for parasphenoid on basioccipital: (0) absent; (1) present.

25. Palatine teeth: (0) absent; (1) present.

26. Number of added plates on epibranchial 1: (0) none; (1) one; (2) two; (3) three; (4) four. (from N26)

27. Large exoccipital foramen: (0) absent; (1) present.

28. Ventral ossification of mesethmoid: (0) absent; (1) present.

29. Pharyngeal process: (0) not-divided medially; (1) divided medially; (2) absent.

30. Process on parasphenoid shaft: (0) no process; (1) oral process. (from N30)

31. Metapterygoid: (0) rectangular; (1) square; (2) rounded; (3) oval; (4) triangular.

32. Number of epurals: (0) two; (1) one.

33. Pterygoid process on palatine: (0) absent; (1) present.

34. Maxillaries: (0) stout; (1) long and narrow.

35. Posterior horizontal process of urohyal: (0) absent; (1) present.

36. Dorsal process on second pharyngobranchial: (0) none; (1) one but bifurcates; (2) two. (from N36)

37. Ventral wing of pelvic plate: (0) short; (1) long.

38. Articulation between pelvic and pectoral girdles: (0) none; (1) postcleithrum to lateral process of pelvic plate; (2) postcleithrum to medial process of pelvic plate; (3) laterally directed pelvic plate to cleithrum.

39. Posterior-most dorsal fin pterygiophore: (0) divided; (1) singular.

40. Alveolar arm of premaxilla: (0) short; (1) intermediate; (1) long. (from N40)

41. Mesethmoid composition: (0) one or two vertical plates; (1) cup with posterior process; (2) one or two horizontal plates.

42. Hypohyal foramen: (0) absent; (1) present.

43. Paired, anterior swelling of swim-bladder: (0) absent; (1) present.

44. Foramen for jugular vein carried over anterior border of exoccipital: (0) no; (1) yes.

45. First ceratobranchial narrowed distally: (0) no; (1) yes.

46. Number of caudal fin rays (branched rays or the ones attached to parhypural and

hypurals): (0) 16; (1) 14; (2) 12. (from N46; *Helostoma* was coded as 1 based on personal observations of OP441; Extant anabantid fishes were coded according to ref. 63)

47. Attachment of Boudelotʼ s ligament on: (0) basioccipital; (1) exoccipital.

48. Orbital process of mesethmoid: (0) absent; (1) present.

49. Ventral longitudinal ridge of frontal joining pterosphenoid to mesethmoid: (0) absent; (1) present.

50. Anterior flange of hyomandibular: (0) broad and angular; (1) broad and rounded; (2) narrow with straight anterior border; (3) small and receding into orbit. (from N50)

51. Supraoccipital with paired posterolateral extensions: (0) absent; (1) present.

52. Preopercular process of quadrate: (0) bifurcated; (1) simple.

53. Number of cephalic lateralis pores in dentary: (0) three; (1) four; (2) canal unossified.

54. Preopercular horizontal arm vs. vertical arm: (0) equal; (1) shorter; (2) longer.

55. Posterior articulation process on epibranchial 1: (0) absent; (1) present.

56. Orientation of posttemporal: (0) in line with supracleithrum; (1) perpendicular to supracleithrum.

57. Teeth on second pharyngobranchial: (0) absent; (1) present; (2) filaments.

58. Caudal peduncle contact organs: (0) absent; (1) present.

59. Anterior hyomandibular flange extends to base of vertical hyomandibular flange: (0) no; (1) yes.

60. Medial process of pelvic plates: (0) short; (1) long and forked; (2) absent.

61. Prop for pelvic-fin spine: (0) absent; (1) present. (from N61, "anal-fin" changed to “pelvic-fin” according to description of ref. 24)

62. Pelvic plates bent anterodorsally: (0) no; (1) yes.

63. Postcranial shelf (from supraoccipital and epioccipital (epiotic)): (0) absent; (1) present.

64. Posterior cephalic lateralis canals in nasals: (0) incompletely enclosed; (1) fully enclosed.

65. Mesethmoid: (0) firmly attached to surrounding elements; (1) loosely attached to surrounding elements.

66. Frontals with depressed and rugous, anteromedial surface: (0) absent; (1) present.

67. Gill rakers of epibranchial 1: (0) absent; (1) present.

68. Fin-spine meristics: (0) high; (1) intermediate; (2) low (6-8); (3) three. (from N68)

69. Pelvic plates: (0) straight and closely set; (1) straight, no internal wings. (from N69)

70. Pointed process on hyomandibular underlying preopercular: (0) absent; (1) present.

71. Metapterygoid with posterior, dorsal process: (0) absent; (1) present.

72. Color pattern on flanks: (0) barred; (1) mottled; (2) blank; (3) spotted.

73. Form of saccular swelling: (0) large and rounded; (1) large and angular. (from N73)

74. Caudal spot: (0) absent; (1) present.

75. Lacrimal (IO1): (0) unserrated; (1) serrated.

76. Posttemporal fossa: (0) absent; (1) present.

77. Parietal, frontal, epioccipital (epiotic) and supraoccipital exposed on inner surface of suprabranchial chamber: (0) no; (1) yes.

78. Mesethmoid contributes to dorsal surface of skull: (0) no; (1) yes.

79. Lateral processes on mesethmoid body: (0) absent; (1) present.

80. Entopterygoid longer than ectopterygoid: (0) no; (1) yes.

81. Infraorbitals 3-5 enclose cheek: (0) no; (1) yes.

82. Ceratohyal foramen: (0) absent; (1) present.

83. Arrangement of branchiostegal rays: (0) two on dorsal (posterior) cereatohyal; (1) one on dorsal (posterior) cereatohyal. (from N83)

84. Ventral medial flange of basihyal: (0) absent; (1) present.

85. Holobranch on fourth ceratobranchial: (0) absent; (1) present.

86. Ventral shaft of ventral postcleithra: (0) stout and cylindrical; (1) narrow and pointed. (from N86)

87. Branchiostegal rays: (0) six: (1) five.

88. Pelvic plates: (0) directed dorsally; (1) lying flat.

89. Ventral plate of mesethmoid: (0) absent; (1) fused to dorsal plate; (2) detached.

90. Frontals: (0) firmly conjoined along entire mesial border; (1) broadly separated anterad.

91. Extrascapulars: (0) divided; (1) singular.

92. Arrangement of supraoccipital commissure: (0) absent; (1) present. (from N92)

93. Suprabranchial chamber: (0) absent; (1) present.

94. Number of posterior openings to trigemino-facialis chamber: (0) three; (1) one.

95. Canals for jugular vein and orbital artery: (0) absent; (1) present.

96. Facet on pterotic for levatoroperculi muscle: (0) absent; (1) present.

97. Pharyngeal process of basioccipital: (0) none; (1) deep. (from N97)

98. First epibranchial expanded into plate: (0) no; (1) yes.

99. Gill filaments across epibranchials: (0) absent; (1) present.

100. Articulation of first pharyngobranchial with prootic: (0) lie against saccular swelling; (1) suspended from anterior prootic.

101. Broad, adjacent articulation surface on pharyngobranchial 3 for third and forth epibranchials: (0) absent; (1) present.

102. Anterior wing of ventral postcleithrum: (0) absent; (1) present.

103. Lachrymal large, triangular or square: (0) no; (1) yes.

104. Pterosphenoids with great anterior extension: (0) no; (1) yes.

105. Posterior extension of swim bladder: (0) absent; (1) present.

106. Square or rectangular opening between metapterygoid and hyomandibualr: (0) absent; (1) present.

107. Ectopterygoid: (0) small; (1) large.

108. Palatine articulates with nasal: (0) no; (1) yes.

109. Articular process of premaxilla: (0) absent; (1) present.

110. Form of interperculars: (0) rectangular; (1) elongate.

111. Urostyle (Compound vertebra 1) fused to hypurals 1 and 2: (0) no; (1) yes.

112. Parasphenoid teeth: (0) absent; (1) present.

113. Medial reach of extrascapular: (0) short; (1) long. (from N113)

114. Ascending process of dentary: (0) none; (1) tall and acute; (2) deep and obtuse. (from N114, *Badis* was coded as 1 according to ref. 68)

115. Mental ossification: (0) absent; (1) present.

116. Uroneural: (0) absent; (1) present.

117. Parhypural: (0) attached; (1) detached but contacting; (2) detached. (from N117)

118. Anal fin spines: (0) absent; (1) present.

119. Form of pharyngobranchial 2: (0) tubular; (1) plate-like; (2) compressed vertical plate.

120. Posterior neurocranium elevated: (0) no; (1) yes.

121. Anterior process of urohyal: (0) absent; (1) present.

122. Orientation or angular/retroarticular: (0) horizontal; (1) vertical.

123. Dentary pivots against angular: (0) no; (1) yes.

124. Anterior extension of urohyal protrudes between ceratohyal bars: (0) no; (1) yes.

125. Palatine articulates with quadrate: (0) no; (1) yes.

126. Anterior border of supraoccipital: (0) straight; (1) acute. (from N126, *Anabas* was coded in state 1 based on observation of specimens OP432-436 (*Anabas testudineus*), not 0 as described by Norris^24^, although the same author coded *Anabas* in state 1 in his own data matrix).

127. Pharyngeal process of parasphenoid: (0) absent; (1) present. (N127)

128. Orientation of symplectic: (0) vertical; (1) horizontal. (N128)

129. Quadrate condyle: (0) directed anteroventrally; (1) directed ventrally. (N129)

130. Articulation cup on operculars: (0) ventral to dorsal border; (1) at dorsal border.

131. Posterior-most anal fin pterygiophore: (0) divided: (1) singular.

132. Supraorbital commissure: (0) present; (1) absent. (from N92)

133. Anteroventral spinous projection of lachrymal: (0) absent; (1) present and articulating with maxilla; (2) present but not articulating with maxilla.

134. The arrangement of openings of infraorbital canal: (0) between infraorbitals; (1)

penetrating infraorbitals.

135. Parietal/pterotic suture length in relative to parietal length: (0) mostly equal; (1)1/2-1; (2) obviously <1/3.

136. Notch on supraoccipital for extrascapular: (0) absent; (1) present.

137. Dorsal fin in relative to anal fin length: (0) obviously longer; (1) nearly equal in length.

138. Breeding behaviour: (0) bubble nesters; (1) free spawners; (2) substrate spawners. (coding according to refs. 25 and 68)

139. Parental care: (0) no parental care; (1) male parental care. (coding according to refs. 25 and 68)

140. Modification of fin rays for courtship rituals: (0) absent; (1) present.

141. Toothed ceratobranchial 5: (0) absent; (1) present. (coding according to refs. 24 and 27)

142. Toothed ceratobranchial 5th biting against transverse process of parasphenoid: (0)

absent; (1) present. (modified from ref. 69, ch.13 in fig. 62)

143. Dentary and premaxillar toothless: (0) absent; (1) present. (modified from ref. 69, ch.15 in fig. 59)

144. Postcleithral ligament: (0) absent; (1) present.

145. Articulation between pelvic plate and cliethrum: (0) present; (1) absent. (from ref. 70)

146. Toothed vomer: (0) present; (1) absent.

147. Number of vertebrae: (0) 24-26; (1) 28; (2) 29. (coding of *Badis* from ref. 68; coding of *C. nebulosum* according to ref. 71 and other living anabantids and *Helostoma* according to ref. 27. Our personal observation of *Anabas testudineus* (OP432-436) counted 28 vertebrae, which is different from the meristics provided in ref. 27)

148. Number of supraneurals: (0) 3; (1) 2; (2) 1-2. (according to ref. 24)

149. Preorbital process of lateral ethmoid for attachment of lachymal: (0) absent; (1) present.

150. Most of anterior dorsal fin pterygiophores approaching anterior neural spines: (0) anteriorly; (1) posteriorly. (according to description in ref. 24)

151. Shape of nasal lateral edge: (0) mostly smooth or straight; (1) triangular; (2) rounded. (according to refs. 24, 27, 68)

152. Type and distribution of scales: (0) ctenoid scales throughout body; (1) cycloid scales on head and ctenoid on trunk; (2) cycloid throughout. (according to ref. 27; condition of *Badis badis* unknown, although other species of *Badis* with cycloid scale on head top and ctenoid scales on trunk^72^).

153. Proportion of length of facet of levator opercula muscle of pterotic length: (0) that facet lacking; (1) <1/3; (2) obviously >1/3.

154. Entire anterior border of opercle articulating with preopercle: (0) no; (1) yes. (from ref. 26)

155. Caudal peduncle: (0) relatively long; (1) short. (according to refs. 23 and 24)

156. Hypural 2 and 3 closely arranged: (0) yes; (1) no.

157. Relationship of anterior anal spines and anal pterygiophores: (0) two anterior spines and one posterior to each pterygiophore; (1) two spines to first pterygiophore; (2) one spine to first anal pterygiophore, all pterygiophores separated; (3) one spine to one anal pterygiophore, all anal pterygiophore fused. (according to ref. 24)

158. Extension of epipleurals to level of caudal fin skeleton: (0) absent; (1) present.

159. Ventral wing in relative to external ventral wing of pelvic plate: (0) short; (1) long.

160. Body shape: (0) oblong; (1) deep-bodied; (2) dwarfed and oblong.

161. Suborbital shelves of infraorbitals: (0) absent; (1) present.

162. Serrations on subopercle: (0) absent; (1) present.

163. Serrations on interopercle: (0) absent; (1) present.

164. Hypural 5’ s shape and size in relative to hypural 4: (0) slender and distinctly much narrower hypural 4; (1) plate-like and largely similar in size with hypural 4.

165. Parhypural size in relative to hypurals 1 and 2 (ventral caudal fin lobe): (0) similar or narrower than hypurals1, 2; (1) distinctly wider than hypurals1, 2.

***Appendix 2***

Datamatrix: Wu et al 2016.nex, Wu et al 2016.tnt, Wu et al 2016 backboned.tnt

**9. Supplementary references, numbering continued from main paper:**

39. Lu, S.-W. *et al*. *Regional Geological Report of Nima Map (H45C001003)(1*: *250000) of the People’s Republic of China* (in Chinese). (Chin. Univ. Geosci., 2010).

40. DeCelles, P. G., Kapp, P., Ding, L., & Gehrels, G. E., Late Cretaceous to mid-Tertiary basin evolution in the central Tibetan Plateau: Changing environments in response to tectonic partitioning, aridification, and regional elevation gain. *Geol. Soc. Am. Bull*. **119**, 654–680 (2007).

41. Kapp, P., DeCelles, P., Gehrels, G., Heizler, M. & Ding, L. Geological records of the Lhasa-Qiangtang and Indo-Asian collisions in the Nima area of central Tibet. *GSA Bull.* **119**, 917–932 (2007).

42. Wang, L.-C., Wang, C.-S., Li, Y.-L., Zhu, L.-D. & Wei, Y.-S. Organic geochemistry of potential source rocks in the Tertiary Dingqinghu Formation, Nima Basin, central Tibet. *J. Pet. Geol.* **34**, 67–85 (2011).

43. Wang, Y.-S. *Regional Geological Survey of the People's Republic of China (Angdar Co)* (in Chinese). (Chin. Univ. Geosci., 2012).

44. Qu, Y.- G., *et al.* *Regional Geological Report of Duoba Map (1*: *250000) of the People’s Republic of China* (in Chinese). (Chin. Univ. Geosci., 2011)

45. Wang, B.-M., Zhou J.-S., Wen, T. & He, Z.-W. Timing of terrestrial strata in Tibetan Nyima Basin and its significance. *Nat. Gas Technol.* **3**, 21–24 (2009).

46. Pei, S., Chen, S., Guo, L., Dransfield, J. & Henderson, A. In *Flora of China Vol. 23* (eds Wu, Z., Raven P. H. & Hong, D.) 132–157 (Beijing: Science Press & St. Louis: Missouri Botanical Garden Press, 2010)

47. Xia, Q.-B. & Lowry II, P. P. in *Flora of China Vol. 13* (eds Wu, Z., Raven, P. H. & Hong, D.) 435–24 (Beijing: Science Press & St. Louis: Missouri Botanical Garden Press, 2007)

48. Xia, N. & Gadek, P. A. in *Flora of China Vol. 12* (eds Wu, Z., Raven, P. H. & Hong, D.) 5–24 (Beijing: Science Press & St. Louis: Missouri Botanical Garden Press, 2007)

49. Xia, N., Turland, N. J. & Gadek, P. A. in *Flora of China Vol. 12* (eds Wu, Z. Raven P. H. & Hong, D.) 1–4 (Beijing: Science Press & St. Louis: Missouri Botanical Garden Press, 2007)

50. Min, T & Barfod, A. in *Flora of China Vol. 11* (eds Wu, Z. Raven P. H. & Hong, D.) 335–357 (Beijing: Science Press and St. Louis: Missouri Botanical Garden Press, 2008)

51. Zhang, Z., Zhang, H. & Endress, P. in *Flora of China Vol. 9* (eds Wu, Z. Raven P. H. & Hong, D.) 18–42 (Beijing: Science Press & St. Louis: Missouri Botanical Garden Press, 2003)

52. Read, R. W. & Hickey, L. J. A revised classification of fossil palm and palm-like leaves. *Taxon* **21**, 129–137 (1972).

53. Wang, Q., Manchester, S. R., Gregor, H., Shen, S. & Li, Z. Fruits of *Koelreuteria* (Sapindaceae) from the Cenozoic throughout the Northern Hemisphere: their ecological, evolutionary, and biogeographic implication. *Am. J. Bot.* **100**, 422–449 (2013).

54. Jia, L. *et al.* First occurrence of *Cedrelospermum* (Ulmaceae) in Asia and its biogeographic implications. *J. Plant Res.* **128**, 747–761 (2015).

55. Ramírez J. L. & Cevallos-Ferriz, S. R. S. A diverse assemblage of Anacardiaceae from Oligocene sediments, Tepexi de Rodriguez, Puebla, Mexico. *Am. J. Bot.* **89**, 535–545 (2002).

56. Wolfe J. Temperature parameters of humid to mesic forests of eastern Asia and relation to forests of other regions of the Northern Hemisphere and Australasia. *Geol. Surv. Prof. Pap. U.S.* **1106**, 1-37 (1979).

57. Collins R. A.， Britz R. and Rüber L. Phylogenetic systematics of leaffishes (Teleostei: Polycentridae, Nandidae), *J. Zoolog. Syst. Evol. Res.* **53**, 259–272 (2015).

58. Murray, A. M., *et al.* A fossil gourami (Teleostei, Anabantoidei) from probable Eocene deposits of the Ombilin Basin, Sumatra, Indonesia, *J. Vert. Paleol.* **35**, 2, e906444 (2015), DOI:10.1080/02724634.2014.906444.

59. Murray, A. M. Relationships and biogeography of the fossil and living

African snakehead fishes (Percomorpha, Channidae, *Parachanna*). *J. Vert. Paleol.* **32**, 820–835 (2008).

60. Das, B. K. The bionomics of certain air-breathing fishes of India, together with an account of the development of their air-breathing organs. *Philos. Trans. R. Soc. Lond. [Biol.]* **216**, 183–219 (1928).

61. Patterson, C. & Johnson, G. D. The intermuscular bones and ligaments of teleostean fishes. *Smithson. Contrib. Zool.* **559**, 1–85 (1995).

62. Gemballa, S. & Britz, R. Homology of intermuscular bones in Acanthomorph fishes. *Am. Mus. Noviates* **3241**, 1–25 (1998).

63. Norris, S. M. & Douglas, M. E. A new species of nest building *Ctenopoma* (Teleostei, Anabantidae) from Zaïre, with a redescription of *Ctenopoma lineatum* (Nichols). *Copeia* **1**, 166–178 (1991).

64. Norris, S. M. *Microctenopoma uelense* and *M. nigricans*, a new genus and two new species of anabantid fishes from Africa. *Ichthyol. Explor. Freshwaters* **6**, 357–376 (1995).

65. Ellis, B. *et al. Manual of leaf architecture* (Cornell Univ. Press, 2009).

66. Skelton, P. H. *A complete guide to the freshwater fishes of southern Africa* (Struik, Cape Town, 2001).

67. Shen, S.-C. *Fishes of Taiwan* (National Taiwan Univ. Press, 1993).

68. Barlow, G., Liem, K. F. & Ickler, W. Badidae, a new fish family-behavioural, osteological, and developmental evidence. *J. Zool. Lond.* **156**, 415–447 (1968).

69. Lauder, G. E. & Liem, K. F. The evolution and interrelationships of the actinopterygian fishes. *Bull. Mus. Comp. Zool*. **150**, 95–197 (1983).

70. Britz, R. Ontogenetic features of *Luciocephalus* (Perciformes, Anabantoidei) with a revised hypothesis of anabantoid intrarelationships. *Zool. J. Linn. Soc.* **112**, 491–508 (1994).

71. Norris, S. M. & Teugels, G. G. A new species of *Ctenopoma* (Teleostei: Anabantidae) from Southeastern Nigeria. *Copeia* **2**, 492–499 (1990).

72. Geetakumari, K. & Kadu, K. *Badis singenensis*, a new fish species (Teleostei: Badidae) from Singen River, Arunachal Pradesh, northeastern India. *J.Threat. Taxa* 3, 2085–2089 (2011).
