# Supplementary material for: Fossil climbing perch and associated plant megafossils indicate a warm and wet central Tibet during the late Oligocene
Source: Sci Rep. 2017 Apr 13;7:878. doi: 10.1038/s41598-017-00928-9 (PMC5429824; doi:10.1038/s41598-017-00928-9)
Supplement: Supplementary file 2 — Dataset 1 [file 41598_2017_928_MOESM2_ESM.doc]

**Data matrix for phylogenetic analysis**

| Character  Taxon |  |
| --- | --- |
| *Badis badis* | 0021002102200100042001100000201100001311000000010101010010010000001300010101?00000111100201101000011110000101001010101101000011001101020021000000011??2?000100?000000 |
| *Helostoma temmincki* | 000000101100000002210110020001310000101021?000000201220020010101000000020011010000111000111110011100001111010101100111111111011100100020110010101122?121?11030?111100 |
| *Anabas testudineus* | 111011211001111110110110021101100111020000000000000111001011000100000010001011001100000110111011110000100010100112012100000001110000210101000100101110201101110011111 |
| *C._multispine* | 01101121111111111012100111011010110210100001100000011100101100010000001000010001001111001011101111000010001010011201210000000111001020010??001001001101111012?1011111 |
| *C._pellegrini* | 01101121111111111012100111011010110210100001100000011100101100010000011000010001001111001011101111000010001010011201210000000111001020010100010010?1101?11012?10111?? |
| *C._nigropannosum* | 01101121111111111012100111011010110210100001100000011100101100010000011000010001001111001011101111000010001010011201210000000111001020010100010010?110???1012?10111?? |
| *C._nebulosum* | 00011101110101111010110104001020100210112110020000011100010010110001001200010001001111001011101111000010001010011201210000000111001020110??001001001112?11111?11110?? |
| *C._petherici* | 10001101110101111012100111001020100210112110000000011100110011010001001210010001001111001011101111000010001010011201210000000111001020110100010010?1112?11111?1111111 |
| *C._kingsleyae* | 10001101110101111012100111001020100?10112110000000011100110011010001001210010001001111001011101111000010001010011201210000000111001020110100010010?1112?11111?11111?? |
| *C._muriei* | 1000110111010111101210011100102010021011211100000001110011001101000100100011000100111100101110111100001000101001120121000000011100102011010001001001112?11111?1011111 |
| *C._weeksii* | 10001101110101111012100101001020100210112110000000011100110011010001001211010001001111001000100010101110111100011201210000000111001020110??0010010?0112?11111?11111?? |
| *C._maculatum* | 10001101110101111012100111001020100210112110000000011100110011010001001211010001001111001011101111000010001010011201210000000111001020100??0010010?1112?11111?11111?? |
| *C._acutirostre* | 00001101110001111012100111001020100210122110000000011100110011010000000310010001001111000111101111000010001010011201210000000111001020100100010010?1112?1111111111111 |
| *C._ocellatum* | 00001101110101111012100111001020101210112110000000011100110011010000000010010001001111001011101111000010001010011201210000000111001020110100010010?1112?11111?11111?? |
| *M._congicum_com.* | 00101001110002110112010103001040100211111001111111100011001100000001010000010011001111001010101111000010001010011201210000000111001120200011010010?1112?21112?1211011 |
| *M._nanum_com.* | 00101001110002110112010103001000100211111001111111100011001100000002001000010011001111001010101111000010001010011201210000000111001120200011010010?1112?11112?1211101 |
| *M._damasi* | 00101001100002110112010103001000100211111001111111100011001100000000001000010011001111001010101111000010001010011201210000000111001120200011010010?1112?11112?12100?? |
| *S._capensis* | 1011110110001111130001011400100110020011211002000101100010110010111110100001000100111110101110111100001000101001120121000000011100102010021001001000112211012?0010011 |
| *S._bainsii* | 2011110110001111130010011400100010020011211002000101100010120010111110100001000100111100100110111100001000101001120121000000011100102010020001001000110211012?0011011 |
| *Eoanabas* | ??00?1?01??10211????101??????0?0?00??111?????0???3??01???00??0?0?0?200????1?????1?1??001?01?1??1?1???01?????1??112?101?0?00??1??0011?00?0??00?0?1?00?121210010?010?00 |
