# Supplementary material for: Fossil climbing perch and associated plant megafossils indicate a warm and wet central Tibet during the late Oligocene
Source: Sci Rep. 2017 Apr 13;7:878. doi: 10.1038/s41598-017-00928-9 (PMC5429824; doi:10.1038/s41598-017-00928-9)
Supplement: Supplementary file 3 — Dataset 2 [file 41598_2017_928_MOESM3_ESM.doc]

xread

165 21

Badis 0021002102200100042001100000201100001311000000010101010010010000001300010101?00000111100201101000011110000101001010101101000011001101020021000000011??2?000100?000000

Helostoma 000000101100000002210110020001310000101021?000000201220020010101000000020011010000111000111110011100001111010101100111111111011100100020110010101122?121?11030?111100

Anabas 111011211001111110110110021101100111020000000000000111001011000100000010001011001100000110111011110000100010100112012100000001110000210101000100101110201101110011111

C._multispine 01101121111111111012100111011010110210100001100000011100101100010000001000010001001111001011101111000010001010011201210000000111001020010??001001001101111012?1011111

C._pellegrini 01101121111111111012100111011010110210100001100000011100101100010000011000010001001111001011101111000010001010011201210000000111001020010100010010?1101?11012?10111??

C._nigropannosum 01101121111111111012100111011010110210100001100000011100101100010000011000010001001111001011101111000010001010011201210000000111001020010100010010?110???1012?10111??

C._nebulosum 00011101110101111010110104001020100210112110020000011100010010110001001200010001001111001011101111000010001010011201210000000111001020110??001001001112?11111?11110??

C._petherici 10001101110101111012100111001020100210112110000000011100110011010001001210010001001111001011101111000010001010011201210000000111001020110100010010?1112?11111?1111111

C._kingsleyae 10001101110101111012100111001020100?10112110000000011100110011010001001210010001001111001011101111000010001010011201210000000111001020110100010010?1112?11111?11111??

C._muriei 1000110111010111101210011100102010021011211100000001110011001101000100100011000100111100101110111100001000101001120121000000011100102011010001001001112?11111?1011111

C._weeksii 10001101110101111012100101001020100210112110000000011100110011010001001211010001001111001000100010101110111100011201210000000111001020110??0010010?0112?11111?11111??

C._maculatum 10001101110101111012100111001020100210112110000000011100110011010001001211010001001111001011101111000010001010011201210000000111001020100??0010010?1112?11111?11111??

C._acutirostre 00001101110001111012100111001020100210122110000000011100110011010000000310010001001111000111101111000010001010011201210000000111001020100100010010?1112?1111111111111

C._ocellatum 00001101110101111012100111001020101210112110000000011100110011010000000010010001001111001011101111000010001010011201210000000111001020110100010010?1112?11111?11111??

M._congicum_comlex 00101001110002110112010103001040100211111001111111100011001100000001010000010011001111001010101111000010001010011201210000000111001120200011010010?1112?21112?1211011

M._nanum_complex 00101001110002110112010103001000100211111001111111100011001100000002001000010011001111001010101111000010001010011201210000000111001120200011010010?1112?11112?1211101

M._ansorgei 00101001100002110112010103001000100211111001111111100011001100000000001000010011001111001010101111000010001010011201210000000111001120200011010010?1112?11112?12100??

M._damasi 00101001110012110112010103001000100211111001111111100011001100000000001100010011001111001010101111000010001010011201210000000111001120200011010010?1112?11112?1210011

S._capensis 1011110110001111130001011400100110020011211002000101100010110010111110100001000100111110101110111100001000101001120121000000011100102010021001001000112211012?0010011

S._bainsii 2011110110001111130010011400100010020011211002000101100010120010111110100001000100111100100110111100001000101001120121000000011100102010020001001000110211012?0011011

Eoanabas ??00?1?01??10211????101??????0?0?00??111?????0???3??01???00??0?0?0?200????1?????1?1??001?01?1??1?1???01?????1??112?101?0?00??1??0011?00?0??00?0?1?00?121210010?010?00

;

cnames

{0 Myodomal_cup absent present_attached_to_ventral_plate_of_mesethmoid no_cup_but_detached_anterior_myodome_bone;

{1 Nasals_conjoined_mesially no yes;

{2 Articulation_of_second_Io_with_La posterad posterodrosad not_at_all;

{3 Lateral_faces_of_Sphenotic_covered_by_pterotics no yes;

{4 Exoccipitals_completely_bound_foramen_magnum no yes;

{5 Supraoccipital_crest present absent;

{6 Epioccipital_process short long_ absent;

{7 Paraspehnoid_shaft straight 'straight,_but_narrowed_at_ascending_wings_';

{8 Transverse_process_of_parasphenoid absent present;

{9 Transverse_process_of_parasphenoid short long no_process;

{10 Terminal_process_of_transverse_process absent present no_process;

{11 Extrascapulars tubular plate-like;

{12 Anterior_flanges_of_Hym_articulates_with_Mpt no yes;

{13 Postmaxillary_process_of_Pmx high low none;

{14 Support_struts_on_medial_side_of_Op single_strut 'V-shaped_struts_' no_struts;

{15 Posterior_notch_on_opercle absent present;

{16 Stout_ligament_joining_paired_fifth_Cbr absent present;

{17 Form_of_valvular_organ multiple_flat_elements_on_Cbr_1_and_2 cupped_structure_ double_cup multiple_flat_elements_on_Cbr_1_only no_valvular_organ;

{18 'Pharyngobrachial_2_in_cross-section_' ovoid round plate;

{19 Valvular_organ none thin_plates bowl-shaped;

{20 Postocular_contact_organ absent present;

{21 Postorbital_process_of_sphenotic absent present;

{22 CLL_pore_at_anterior_border_of_pterotic absent present;

{23 Medial_descending_process_for_Psp_on_Basioccipital absent present;

{24 Palatine_teeth absent present;

{25 Number_of_added_plates_on_epibranchial_1 none one two three four;

{26 Large_exoccipital_foramen absent present;

{27 Ventral_ossification_of_mesethmoid_ absent present;

{28 Pharyngeal_process_of_Basioccipital 'not-divided_medially_' divided_medially absent;

{29 Process_on_Psp_shaft no_process oral_process;

{30 Metapterygoid rectangular square rounded oval triangular;

{31 Number_of_epurals two one;

{32 Pterygoid_process_on_palatine absent present;

{33 Maxillaries stout long_and_narrow;

{34 Posterior_horizontal_process_of_urohyal absent present;

{35 Dorsal_process_on_Pbr_2 one one_but_bifurcated two;

{36 Ventral_wing_pelvic_plate short long;

{37 Articulation_between_pelvic_and_pectorlal_girdle none Pscl_to_lateral_process_of_pelvic_plate Pscl_to_medial_pelvic_plate laterally_directed_pelvic_plate_to_Cl;

{38 'Posterior-most_dorsal-fin_pterygiophore_' divided singular;

{39 Alveolar_process_of_Pmx short intermediate long;

{40 Mesethmoid_composition one_or_two_vertical_plates cup_with_posterior_process one_or_two_horizontal_plates;

{41 Hypohyal_foramen absent present;

{42 'Paired,_anterior_swellings_of_swim-bladder_' absent present;

{43 Foramen_for_jugular_vein_carried_over_anterior_border_of_exoccipital no yes;

{44 Cbr_1_narrowed_distally no yes;

{45 'Number_of_caudal_fin_rays_(branched)_' 16 14 12;

{46 Attachment_of_Baudelot_s_ligament_on basioccipital exoccipital;

{47 Orbital_process_of_mesethmoid_ absent present;

{48 Ventral_longitudinal_ridge_of_Fr_joining_pterospenoid_to_mesethmoid absent present;

{49 Anterior_flange_of_Hym broad_and_angular broad_and_rounded narrow_with_straight_anterior_border small_and_receding_into_orbit;

{50 Supraoccipital_with_paired_posterolateral_extensions absent present;

{51 Preopercular_process_of_Qu bifurcated simple;

{52 Number_of_cephalic_lateralis_pores_in_dentary three four canal_unossified;

{53 Preopercular_horizontal_arm_vs_vertical_arm equal shorter longer;

{54 Posterior_articulation_process_on_epibranchial_1 absent present;

{55 Orientation_of_Pt in_line_with_Scl perpendicular_with_Scl;

{56 Teeth_on_Pbr_2 absent present filament;

{57 Caudal_peduncle_contact_organ absent present;

{58 Anterior_flange_of_Hym_extends_to_base_of_vertical__Hym_shaft no yes;

{59 Medial_process_of_pelvic_plates short long_and_forked_ absent;

{60 'Prop_for_pelvic-fin_spine_' absent present;

{61 Pelvic_plates_bent_anterodorsally no yes;

{62 'Postcranial_shelf_(from_supraoccipital_and_epioccipital))_' absent present;

{63 Posterior_cephalic_lateralis_canals_in_nasals incompletely_enclosed fully_enclosed;

{64 Mesethmoid_ firmly_attached_to_surrounding_elements loosely_attached_to_surrounding_elements;

{65 'Frontals_with_depressed_and_rigous,_anteromedial_surface_' absent present;

{66 Gill_rakers_of_epibranchial_1 absent present;

{67 'Fin-spine_meristics_' high intermediate low three;

{68 Pelvic_plates straight_and_closely_set 'straight,_no_internal_wings_';

{69 Pointed_process_on_Hym_underlying_Pop absent present;

{70 'Metapterygoid_with_posterior,_dorsal_process_' absent present;

{71 Color_patterns_on_flanks barred mottled blank spotted;

{72 Form_of_saccular_swelling large_and_rounded large_and_angular;

{73 Caudal_spot absent present;

{74 'Lachrymal_(Io_1)_' unserrated serrated;

{75 Posttemporal_fossae absent present;

{76 'Parietal,_frontal,_epioccipital_and_supraoccipital_exposed_on_inner_surface_of_Suprabr._chamber_' no yes;

{77 Mesethmoid_contributes_to_dorsal_surface_of_skull_table no yes;

{78 Lateral_processes_on_mesethmoid_body absent present;

{79 Entopterygoid_longer_than_ectopterygoid no yes;

{80 'Io_3-5_enclose_cheek_' no_ yes;

{81 Ceratohyal_foramen absent present;

{82 Arrangement_of_Br_rays two_on_dorsal_ceratohyal one_on_dorsal_ceratohyal;

{83 Ventral_medial_flange_of_basihyal absent present;

{84 Holobranch_on_Cbr_4 absent present;

{85 Ventral_shalft_of_ventral_postcleithra stout_and_cylindrical narrow_and_pointed;

{86 Br_rays 6 5;

{87 Pelvic_plates directed_dorsally lying_flat;

{88 Ventral_plate_of_mesethmoid_ absent fused_to_dorsal_plate detached;

{89 Frontals firmly_conjoined_along_entire_mesial_border broadly_separated_anterad;

{90 Extrascapulars divided singular;

{91 Supraoccipital_commisure_ absent present;

{92 Suprabranchial_chamber absent present;

{93 'Number_of_posterior_openings_of_Trig-Fac_chamber_' three one;

{94 Canals_for_jugular_vein_and_orbital_artery absent present;

{95 Facet_on_pterotic_for_levator_operculii_muscle absent present;

{96 Pharyngeal_process_of_basioccipital none deep;

{97 Epibr._1_expanded_into_plate no_ yes;

{98 Gill_filaments_across_epibr. absent present;

{99 Articulation_of_Phybr._1_with_prootic lies_against_saccular_swelling suspended_from_anterior_prootic;

{100 'Broad,_adjacent_articulation_surface_on_Phbr._3_for_Epibr._3_and_4_' absent present;

{101 Anterior_wing_of_ventral_Postcleithrum absent present;

{102 'Lachymals_large,_triangular_or_square_' no yes;

{103 Pterosphenoids_with_great_anterior_extension no yes;

{104 Posterior_extension_of_swim_bladder absent present;

{105 Square_or_rectangular_opening_between_Mpt_and_Hym_or_Qu? absent present;

{106 Ectopterygoid small large;

{107 Palatine_articulates_with_nasal no yes;

{108 Articular_process_of_Pmx absent present;

{109 Form_of_Interopercle rectangular elongate;

{110 'Urostyle_fused_to_hypurals_1(6)_and_2_(5)_' no yes;

{111 Parasphenoid_teeth absent present;

{112 Medial_reach_of_extrascapular_ short long;

{113 Ascending_process_of_dentary none tall_and_acute deep_and_obtuse;

{114 Mental_ossification absent present;

{115 Uroneural_ absent present;

{116 Parhypural_with_the_urostyle attached detached_but_contacting detached;

{117 Anal_fin_spines absent present;

{118 Form_of_Phbr._2 tubular plate-like compressed_vertical_plate;

{119 Posterior_neurocranium_elevated no yes;

{120 Anterior_process_of_urohyal absent present;

{121 'Orientation_of_angular/retroarticular_' horizontal vertical;

{122 Dentary_pivots_against_angular no yes;

{123 Anterior_extension_of_urohyal_protrudes_detween_ceratohyal_bars no yes;

{124 Palatine_articulates_Qu no yes;

{125 Anterior_border_of_supraoccipital straight acute;

{126 Pharyngeal_process_of_Psp absent present;

{127 Orientation_of_symplectic vertical_ horizontal;

{128 Quadrate_condyle directed_anteroventrally directed_ventrally;

{129 Articulation_cup_on_Op ventral_to_dorsal_border at_dorsal_border;

{130 'Posterior-most_anal-fin_pterygiophore_' divided singular;

{131 Supraorbital_comm present absent;

{132 Anteroventral_spinous_projection_of_lachrymal absent present_and_articulating_with_maxilla presetn_but_not_articulating_with_maxilla;

{133 The_opening_of_infraorbital_canal_in_infraorbitals inbetween_Io penetrating_Io;

{134 'Parietal/Pterotic_suture/_Parietal_axial_length_' ca._1 '1/2_to_1' 'obviously_<1/3_';

{135 Notch_on_supraoccipital_for_extrascapular absent present;

{136 Dorsal_fin_in_relative_to_anal_fin_length obviously_longer nearly_equal_in_length;

{137 Breeding_behavior bubble_nesters free_spawner substrate_spawner;

{138 Parental_care no male_parental_care;

{139 Modification_of_fin_rays_for_courtship_rituals absent present;

{140 Toothed_Cbr_5 present absent;

{141 Toothed_Cbr5_biting_against_transverse_pr_of_Psph absent present;

{142 Dentary_and_premaxillar_toothless absent present;

{143 Postcleithral_ligament present absent;

{144 Articulation_between_pelvic_plate_and_cliethrum present absent;

{145 Toothed_vomer present absent;

{146 Number_of_vertebrae 24-26 28 29;

{147 'Number_of_supraneural_(predorsal)_' 3 2 1-2;

{148 Preorbital_process_of_lateral_ethmoid_for_atachment_of_Lachymal absent present;

{149 Dorsal_fin_pterygiophore_approaching_neural_spines anteriorly posteriorly;

{150 Shape_of_nasal_lateral_edge mostly_smooth_or_straight triangular rounded;

{151 Type_of_scales ctenoid_scales_throughut cycloid_on_head_top_and_ctenoid_on_body cycloid_throughout_;

{152 Proportion_of_facet_on_levator_operculi_m._of_pterotic_length none <1/3 >1/3;

{153 Entire_anterior_border_of_Opercle_articulating_with_preopercle no yes;

{154 Caudal_peduncle relatively_long short;

{155 Hypural_2_and_3_closely_arranged yes no;

{156 Relationship_of_anterior_anal_spines_and_anal_pterygiophores two_anterior__spines_and_one_posterior_to_each_pterygiophore two_to_first_pterygiophore one_spine_to_one_anal_pterygiophore 'one_spine_to_one_anal_pterygioephore,_all_anal_pterys_fused';

{157 Extension_of_epipleurals_to_level_of_caudal_fin_skeleton absent present;

{158 Ventral_wing_in_relative_to_external_ventral_wing_of_plevic_plate short long;

{159 Body_shape_ Oblong Deep-bodied Dwarfed_and_oblong;

{160 Suborbital_shelf_on_infraorbitals absent present;

{161 Serrations_on_subopercle absent present;

{162 Serrations_on_interopercle absent present;

{163 Hypural_5_shape_and_size_in_relative_to_hypural_4 slender_and_distinctly_much_narrower_than_hypural_4 'plate-like_and_larhely_similar_in_size';

{164 'Size_of_Parhypural_relative_to_hypurals_1,_2' 'parhypurals_smaller_than_hypurals1,_2' 'parhypurals_larger_than_hypurals_1,_2';

;

proc /;

comments 0

;
